# Large-scale phage-based screening reveals extensive pan-viral mimicry of host short linear motifs

**DOI:** 10.1038/s41467-023-38015-5

**Published:** 2023-04-26

**Authors:** Filip Mihalič, Leandro Simonetti, Girolamo Giudice, Marie Rubin Sander, Richard Lindqvist, Marie Berit Akpiroro Peters, Caroline Benz, Eszter Kassa, Dilip Badgujar, Raviteja Inturi, Muhammad Ali, Izabella Krystkowiak, Ahmed Sayadi, Eva Andersson, Hanna Aronsson, Ola Söderberg, Doreen Dobritzsch, Evangelia Petsalaki, Anna K. Överby, Per Jemth, Norman E. Davey, Ylva Ivarsson

**Affiliations:** 1 Department of Medical Biochemistry and Microbiology, Uppsala University, Box 582, Husargatan 3, 751 23 Uppsala, Sweden; 2 Department of Chemistry - BMC, Uppsala University, Box 576, Husargatan 3, 751 23 Uppsala, Sweden; 3 European Molecular Biology Laboratory-European Bioinformatics Institute, Hinxton, CB10 1SD UK; 4 Department of Pharmaceutical Biosciences, Uppsala University, Husargatan 3, Box 591, SE-751 24 Uppsala, Sweden; 5 Department of Clinical Microbiology, Umeå University, 90187 Umeå, Sweden; 6 Laboratory for Molecular Infection Medicine Sweden (MIMS), Umeå University, 90186 Umeå, Sweden; 7 Division of Cancer Biology, The Institute of Cancer Research, 237 Fulham Road, London, SW3 6JB UK

**Keywords:** Protein-protein interaction networks, Viral proteins, Peptides

## Abstract

Viruses mimic host short linear motifs (SLiMs) to hijack and deregulate cellular functions. Studies of motif-mediated interactions therefore provide insight into virus-host dependencies, and reveal targets for therapeutic intervention. Here, we describe the pan-viral discovery of 1712 SLiM-based virus-host interactions using a phage peptidome tiling the intrinsically disordered protein regions of 229 RNA viruses. We find mimicry of host SLiMs to be a ubiquitous viral strategy, reveal novel host proteins hijacked by viruses, and identify cellular pathways frequently deregulated by viral motif mimicry. Using structural and biophysical analyses, we show that viral mimicry-based interactions have similar binding strength and bound conformations as endogenous interactions. Finally, we establish polyadenylate-binding protein 1 as a potential target for broad-spectrum antiviral agent development. Our platform enables rapid discovery of mechanisms of viral interference and the identification of potential therapeutic targets which can aid in combating future epidemics and pandemics.

## Abstract (web summary)

Protein-protein interactions underlie all aspects of a viral infection. Here the authors employ a pan-viral approach for systematic identification of motif-mediated interactions between viral and human proteins and show that the information can be used to find targets for antiviral drug development.

## Introduction

Viruses are obligate intracellular parasites that depend on the host cell machinery for successful infection and replication^1^. As such they hijack and deregulate the host cell machinery through virus-host protein–protein interactions (PPIs) that often involve interactions between folded host proteins and viral short linear motifs (SLiMs)^2,3^. SLiMs are compact and degenerate protein interaction modules, typically encoded in protein regions between three to ten amino acids in length and often, but not always, found in intrinsically disordered regions (IDRs) of proteins^4,5^. Viral proteins have convergently evolved SLiMs that mimic host SLiMs to outcompete endogenous interactions and to rewire host networks to the advantage of the virus^2,3^. Such SLiM-based hijacking has been reported for all stages of viral infection, including viral cell entry, replication, assembly, release, and subversion of the cellular defense response^2,6^. Mimicry of host SLiMs provides viruses with an elegant solution to the spatial constraints of their genomes as compact SLiM interfaces allow for high functional density within a limited protein region.

Virus-host PPIs have been mapped for several viruses through affinity purification-mass spectrometry (AP-MS) and yeast two-hybrid (Y2H) based approaches^7–12^. In addition, more than 200,000 virus-host PPIs have been suggested from computational structure-based pan-viral analyses^13^. However, SLiM-based interactions are likely underrepresented in the available large-scale virus-host PPI datasets because the methods used are not optimized to capture low-affinity transient SLiM-based interactions^14,15^. Consequently, most SLiM-based virus-host PPIs have been identified using low-throughput methods^4^. Nevertheless, bioinformatic analysis has suggested that viral mimicry of host SLiMs is a common strategy for viral takeover^16^, and many questions remain to be answered by systematic and unbiased pan-viral studies. For example, it is not clear how pervasive the viral use of SLiM-based interactions is, what similarities and differences exist among viral families in terms of preferred host targets, and to what extent virus-host PPIs converge upon specific vulnerabilities in the hosts networks.

In this study, we present an extensive pan-viral dataset of interactions between viral motifs and human protein domains generated by proteomic peptide phage display (ProP-PD) using a phage library containing peptides from 229 RNA viruses and 139 human bait protein domains^17^. Based on our results we (i) show that most viruses mimic host SLiMs to interact with host proteins, (ii) identify weak points in cellular pathways that are susceptible to viral interference, (iii) demonstrate that the IDRs of many viral proteins contain multiple overlapping or adjacent SLiMs highlighting high functional density, (iv) show how viral SLiMs can exploit endogenous PPIs by binding host domains with comparable affinities to endogenous ligands, and (v) demonstrate how our approach can identify potential targets for the development of novel antiviral agents.

## Results

### Large-scale screening using an RNA virus peptidome reveals ubiquitous pan-viral SLiM-based mimicry

**Fig. 1:**
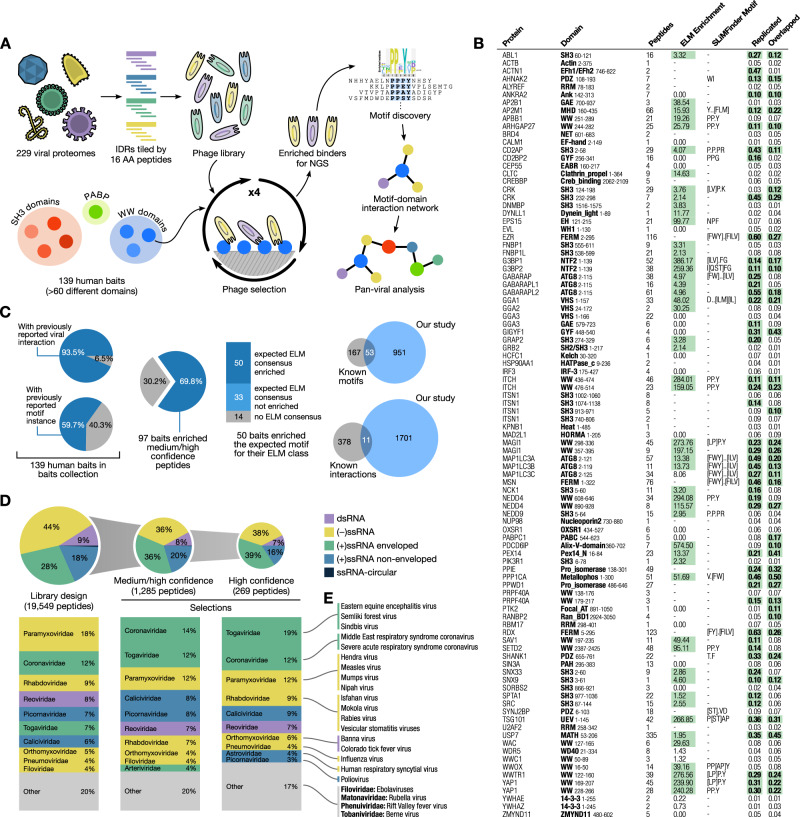
Overview of the RiboVD library design and selection outcome. **A** General workflow of the ProP-PD selection process and the data analysis. **B** Overview of the RiboVD selection results showing for each bait the number of enriched medium/high confidence peptides, the enrichment of peptides with sequences matching the consensus motif reported in ELM database, the generated motifs based on the enriched peptides (SLiMFinder Motif), and the quality of the selection results. “Replicated” represents the proportion of all peptides found in selections that were replicated in independent selections with the same bait while “Overlapped” is the proportion of all peptides that had overlapping peptides among all replicates for each bait. Green highlight indicates high quality of results. **C** Overview of the bait collection used in this study and the comparison of the selection results in context of previously known information. Left, percentage of baits used with previously known viral interactors (130 out of 139, Supplementary data 2) or previously reported motif instances (83 out of 139, Supplementary data 4). Center, percentage of bait domains that enriched peptides in selections and how their enriched motifs (if any) relate to the 83 motif instances previously reported in ELM. Right, overlap between the number of interactions in the RiboVD selections results and previously reported human-virus SLiM-based interactions (Supplementary data 4), or human-virus PPIs (Supplementary data 5). **D** RiboVD library composition and peptide distribution before and after selections. Represented as pie-plots are the percentages of RiboVD peptides belonging to the five different RNA viral genome types before (library design) and after selections. Below the pie charts are the top 10 viral families present in each pool. The representation of peptides for viruses with ssRNA-circular genomes was 103 peptides (0.5%) for the RiboVD library design, and 3 peptides (0.2%) for the medium/high confidence peptide set. **E** Examples of viruses from different virus families investigated in this study.

We screened for virus-host interactions using a previously described phage display library that displays the IDRs from 229 RNA viruses on the major coat protein P8 of the filamentous M13 phage^17^. This Riboviria Viral Disorderome (RiboVD) library (Supplementary data 1; 19,549 unique 16 amino acid-long peptides; 96.4% confirmed by next-generation sequencing (NGS; Figure S1)) contains an almost equal contribution of peptides from positive-sense single-stranded RNA ((+) ssRNA) and negative-sense ss RNA ((−) ssRNA) viruses. A minor fraction of the peptides originated from double-stranded (ds) RNA viruses and a very small percentage of the peptides are from the *Hepatitis delta virus,* which is a circular ssRNA virus (Fig. 1D). The *Paramyxoviridae* family ((−) ssRNA) contributed with most of the peptides to the library design, followed by the *Coronaviridae* ((+) ssRNA) and the *Rhabdoviridae* ((−) ssRNA) families. Viral families with lesser contribution of peptides were for example *Flaviviridae* ((+) ssRNA; 288 peptides) and *Bornaviridae* ((−) ssRNA; 86 peptides). The differences in the peptide distribution arise from variation in the availability of sequence information for different viral families, as well as length and intrinsic disorder content of the viral proteomes.

Using the RiboVD library, we performed triplicate ProP-PD selections against 139 human bait protein domains (Fig. 1A, B; Supplementary data 2), representing more than 60 different domain families. The bait protein domains were selected to include domains from proteins that have prior reports of interactions with SARS-CoV-2 proteins^18^, and also included protein domains that are known to interact with SLiMs and are efficiently expressed in *E. coli*^14,19^. Some of these proteins have previously been reported to bind to viral SLiMs, e.g., WW domains^20,21^, SRC homology 3 (SH3) domains^22,23^ and protein phosphatase 1 (PPP1CA)^24^. Immobilized bait proteins were challenged with the RiboVD library, unbound phages were washed away, and bound phages were eluted and amplified for the next round of selection. The enrichment of binding phages was evaluated by enzyme-linked immunosorbent assay, and the sequences of binding-enriched phage pools were analyzed by NGS. Confidence levels were assigned for the identified peptides based on previously defined quality metrics, namely if they were (i) re-discovered in replicate selections against the same bait protein, (ii) highly enriched during selections, (iii) containing a consensus motif or (iv) if the motif was found with overlapping peptides^14^. For benchmarking of quality metrics when applied to RiboVD see Figure S2. In total, we identified 1285 medium/high confidence viral peptide ligands binding to 97 domains that fulfilled at least two of the quality metrics^14^ (Supplementary data 3; Fig. 1B). Virus-derived peptides binding to host protein domains were found for nearly 90% of the viral species present in the library, covering all 26 represented viral families. After the selections, there was a shift in the distribution of peptides towards peptides from (+) ssRNA viruses (Fig. 1D), which may indicate a difference in motif-density between (−) and (+) ssRNA viruses.

To assess the extent to which the RiboVD selections re-discovered known cases of viral motif mimicry we generated a RiboVD motif benchmarking set (Supplementary data 4) which included interactions collected from the Eukaryotic Linear Motif (ELM) database^4^, interaction pairs extracted from the Protein Data Bank (PDB), manually curated information from the literature and putative interactions generated by incorporating data from homologous domains. Notably, the interactions in the RiboVD motif benchmarking set were found using a variety of approaches (e.g. peptide arrays, phage display of domains, low-throughput pulldown experiments, and viral assays). Of 220 viral SLiMs from the benchmarking set that were present in the RiboVD library, 53 were re-discovered by the selections (Fig. 1C; Supplementary data 4). The motif-rediscovery rate (24% recall) was high, surpassing our recent benchmarking results for a human disorderome phage library (19.3% rediscovery)^14^. We further compiled a virus-host PPI reference set based on data available in IntAct^25^, BioGrid^26^, VirHostNet^27^ and other published sources (Supplementary data 5). The virus-host PPI reference set contained 389 virus-host PPIs involving proteins used in our study, interactions that could thus potentially be found in our study. However, only 11 of the interactions (2.8%) in the virus-host PPI reference set were found by the RiboVD selections. Several factors contribute to the limited overlap. Most of the interactions in the virus-host PPI reference set were reported based on AP-MS or proximity-labeling coupled to MS approaches, methods that report on binary interactions but also on larger complexes as well as proximity to the bait protein. Thus, these datasets have lower proportions of direct binary interactions that can be discovered in our experiments. In addition, there is a bias for more stable interactions in pulldown approaches, which likely contributes to the limited overlap. Finally, it should be noted that we used isolated domains, and not full-length proteins, as baits to generate the RiboVD data. Consequently, the RiboVD selections cannot identify interactions mediated by other parts of the proteins. Taken together, there are several underlying reasons that contribute to the low overlap between the RiboVD data and the virus-host PPI reference set.

### Viral motifs bind to common and distinct host targets

**Fig. 2:**
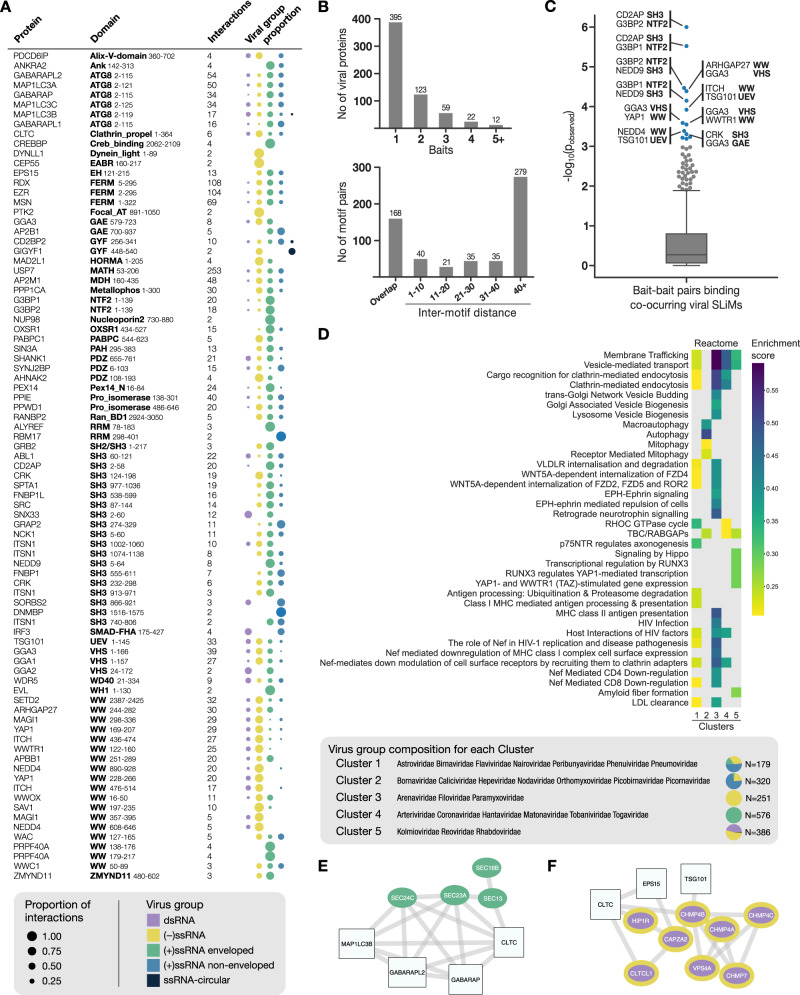
Viral-host PPI and network analysis. **A** Overview of the interactions identified per bait, together with the distribution of ligands from different types of viruses. **B** Viral targeting of bait proteins and motif proximity. Top: Number of screened baits recognized per viral protein showing that over 200 viral proteins bind to 2 or more human bait proteins. Bottom: Distribution of co-occurring viral motif-motif distances (in amino acids) showing that about a third of the viral peptides (208 out of 578) are 10 or less amino acids apart with most of them directly overlapping with each other. **C** Analysis of co-occurrence of host-binding peptide ligands in viral proteins (that is, peptides located in the same viral protein binding distinct bait domains) and their binding to protein domains. The *p*-values of the enrichment of co-occurring peptide ligands targeting human bait-bait pairs are shown (Supplementary data 6). Each dot represents a unique human bait-bait pair being targeted by co-occurring viral peptide ligands. Bait-bait pairs with *p* < 0.001 (based on randomization) are highlighted in blue and the top 10 human bait-bait pairs are labeled. **D** Clustering of host hijacking network signatures revealed five groups enriched in similar and distinct Reactome pathways. The relative frequency represents the enrichment score adjusted to account for the number of members in each viral family that are contributing to the enrichment. The N next to the pie charts indicates the number of identified interactions for all viruses in the cluster. **E** Sub-network of the COPII complex components (green) identified for cluster 4 based on network diffusion, together with their first neighbor bait proteins used in the RiboVD screen. **F** Sub-network of the ESCRTIII components (purple with yellow border) identified for cluster 5 based on network diffusion, together with their first neighbor bait proteins used in the RiboVD screen.

The results of the RiboVD selections provided extensive pan-viral information on virus-host PPIs, which allowed us to analyze the relationship between the viral phylogeny and the type of host proteins they interact with (Fig. 2A). We observed that while some proteins were targeted by specific groups of viral species (e.g., ALYREF RRM and PRPF40A WW by (+) ssRNA viruses), the data pointed towards a broad distribution of viral families binding specific baits (e.g., USP7 MATH and WDR5 WD40) indicating large overlaps of the viral SLiM-mediated interactomes. The results allowed the exploration of the molecular interplay between distinct types of viral SLiMs (Fig. 2B, C). While close to 400 viral proteins bound to a single bait protein, over 200 viral proteins contained more than one type of SLiM. In most cases, these co-occurring motifs were found distal in the amino acid sequence, such that they can interact with their binding partners independently. However, 208 out of 578 co-occurring motifs overlapped or were in close proximity (1–10 amino acids), implying that the motifs compete with each other for binding to distinct host proteins (Fig. 2B).

A subset of SLiMs co-occurred more frequently in viral proteins than would be expected by chance (Fig. 2C). For example, the NTF2 domains of the Ras GTPase-activating protein-binding proteins 1 and 2 (G3BP1/2) and the SH3 domains of the CD2-associated protein (CD2AP) interact with co-occurring SLiMs in the non-structural protein 3 (Nsp3) of several alphaviruses (*Togaviridae)*. These motifs are located distal in sequence and both G3BP1/2 and CD2AP have previously been shown to interact with Nsp3 and to co-localize with viral replication complexes in alphaviruses^28,29^. An example of co-occurring overlapping motifs is provided by SLiMs binding to the E3 ubiquitin-protein ligase NEDD4 WW domain (NEDD4 WW) and the tumor susceptibility gene 101 protein UEV domain (TSG101 UEV). The TSG101 and NEDD4 WW binding motifs enable viral egress by hijacking the endosomal sorting complexes required for transport (ESCRT) machinery^30^. These SLiMs were found to predominantly co-occur in enveloped (−) ssRNA viruses such as Rabies virus (RABV; *Rhabdoviridae*) and Ebola virus (EBOV; *Filoviridae*). Competitive binding between NEDD4 WW and TSG101 UEV binding motifs have been reported for the EBOV viral matrix protein VP40^31^, and they were also found in our study. We further found overlapping co-occurring WW and TSG101 UEV binding motifs in the Nsp3 of Bluetongue virus, interactions that have been validated elsewhere^32^ although the competition between the motifs was not previously discussed.

### Clustering of host target networks reveals network signatures of viral hijacking

To pinpoint host processes that are commonly targeted by viruses beyond the interactions identified by the RiboVD selections, we used a network diffusion approach. Such analysis assumes that if a human protein is targeted by viral proteins, its neighboring proteins in a protein interaction network are also likely to be important for and/or affected by viral hijacking. Thus, if multiple host proteins fall in a similar region of the network, network modules or signatures relevant to viral hijacking will be highlighted. This analysis allowed us to extract network signature perturbations for each virus in the dataset. Functional enrichment analysis of these signatures revealed that RNA viruses preferentially target proteins involved in protein transport, in particular endocytosis, autophagy, cell morphogenesis, and cell signaling (Figure S3; Supplementary data 7). Next, we searched for network modules or processes that were unique to specific virus types. We clustered the viral families according to their interaction networks and identified five main clusters (Fig. 2D; Figure S4). While cluster 1 was heterogeneous, the other four clusters were dominated by distinct types of viruses: cluster 2: mostly non-enveloped (+) ssRNA viruses, cluster 3: enveloped (−) ssRNA viruses, cluster 4: enveloped (+) ssRNA viruses, and cluster 5: dsRNA viruses and (−) ssRNA viruses. All viruses except those in cluster 2 targeted processes related to vesicle-mediated transport, with the enveloped (−) ssRNA and (+) ssRNA viruses in cluster 3 and 4 targeting clathrin-mediated endocytosis (Fig. 2D). For (−) ssRNA viruses we also observed an enrichment of proteins involved in Golgi associated vesicular budding. In contrast, for the non-enveloped viruses and the enveloped (−) ssRNA viruses (*Orthomyxoviridae* and *Bornaviridae*) in cluster 2 there was an enrichment of processes associated with autophagy, through direct interactions with the ATG8-like host proteins (microtubule-associated proteins 1A/1B light chain 3 (MAP1LC3s) and gamma-aminobutyric acid receptor-associated proteins (GABARAPs)). The distinct signature for cluster 2 may be related to the fact that non-enveloped viruses do not require trafficking machinery for lytic release but instead use autophagy for non-lytic egress^33–35^. Some viruses in cluster 2 such as poliovirus (*Picornaviridae*) have also been reported to use the autophagy machinery during early replication events^36,37^. Furthermore, the enveloped *Orthomyxoviridae viruses* (e.g., Influenza A) in cluster 2 bud at the plasma membrane independent of the ESCRT machinery^38^.

Overall, there are both similarities and differences in functional enrichments between the different clusters (Fig. 2D), consistent with hijacking of similar processes but also with distinct signatures of host network interference between different viral groups. For example, comparing the enriched proteins involved in vesicle-mediated transport between the (+) ssRNA viruses in cluster 4 (e.g., *Coronaviridae*) and the (−) ssRNA and dsRNA viruses in cluster 5 (e.g., *Rhabdoviridae* and *Reoviridae*), we found that the former are enriched in proteins linked to the cytoplasmic coat protein complex II (COPII), which sorts cargo from the endoplasmic reticulum (ER) to the trans-Golgi network^39^, while the latter are enriched in proteins associated with the ESCRT-III complex involved in reverse topology vesicular egress and viral budding (Fig. 2C; Figure S5)^30^. Coronaviruses (in cluster 4) assemble by budding into the lumen of the intermediate compartment at the ER-Golgi interface^40^. In contrast, members of the *Rhabdoviridae* family (cluster 5; e.g., RABV and vesicular stomatitis virus (VSV)) bud at the plasma membrane via the ESCRT complex^30^. The result may thus be linked to differences in budding between the distinct viral clusters.

To demonstrate how our RiboVD data can provide deeper insights, we selected protein interactions involved in three biological processes (the ESCRT machinery, endocytosis, and protein translation) for detailed investigation.

### Hijacking of the ESCRT machinery highlights motif co-occurrence

**Fig. 3:**
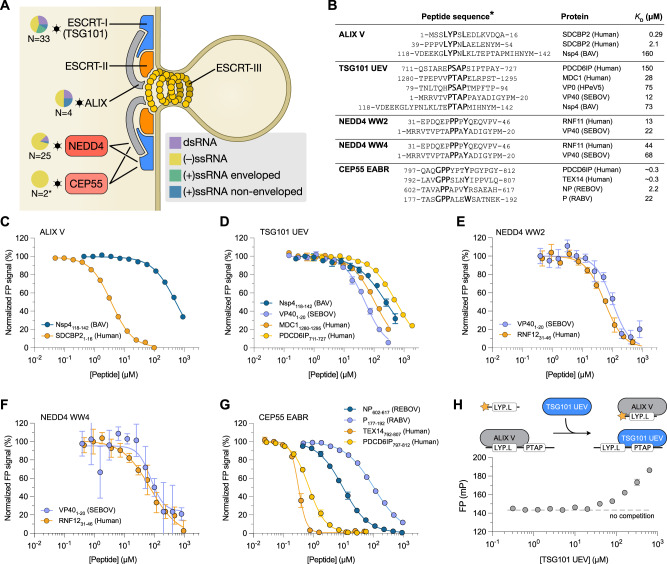
The ESCRT machinery is hijacked by viral SLiMs that bind to NEDD4 WW, TSG101 UEV and ALIX V, and potentially also to CEP55 EABR. **A** Schematic representation of the ESCRT pathway leading to reverse topology budding. Pie charts next to each target show the class of viral species hijacking it. N represents the number of identified interactions. **B** Overview of the peptides that bind to ESCRT pathway proteins for which affinities were measured. Residues constituting the recognition motif are shown in bold. **C**–**G** FP-monitored displacement experiments of viral and human peptides and ESCRT proteins. All FP-monitored experimental data in this paper are represented as normalized means ± SD of at least three replicate experiments. For detailed information on the peptides used in this study see Supplementary data 8. For all FP affinity measurements performed in this study see Figure S6. Source data for these and all subsequent FP-monitored measurements are provided in Source data files. **H** FP-monitored displacement experiment of Nsp4_118-142_ (BAV) shows that the interaction with ALIX V and TSG101 UEV is mutually exclusive.

Many viruses exploit the ESCRT pathway machinery for viral budding by binding to the TSG101 UEV domain, the WW domains of NEDD4 and the V domain of programmed cell death 6-interacting protein, commonly called ALIX (Fig. 3A). These interactions facilitate nuclear envelope budding, formation of double-membrane replication complexes and egress of viral particles from the host cell membranes^30,41^. Selections against the three aforementioned ESCRT-related proteins resulted in 81 peptide hits from 12 virus families, most of them from (−) ssRNA viruses. In addition, we identified interactions between the ESCRT-associated centrosomal protein of 55 kDa EABR domain (CEP55 EABR) and the Reston ebolavirus (REBOV) nucleoprotein (NP), as well as the RABV protein P (Fig. 3B, G). CEP55 interacts with TSG101 and ALIX to form a complex that is involved in abscission of the plasma membrane at the midbody during cell division^42^.

We determined affinities for ALIX V, NEDD4 WW, TSG101 UEV, and CEP55 EABR with viral and human peptides using a fluorescence polarization (FP) based assay (Fig. 3B–G; Figure S6; Supplementary data 8). The human reference ligands used were previously reported in the ELM database^4^ (the TSG101 binding peptide from ALIX (PDC6IP); the CEP55 binding peptides from ALIX^43^ and TEX1^44^) and/or previously found as interactors through ProP-PD (the ALIX V domain binding peptides from SDCBP2 and MDC40, and the NEDD4 binding peptide from RNF11)^14^. Notably, the RNF11-NEDD4 interaction has been reported also by others^45^. Furthermore, the ALIX V binding SDCBP2 peptides share high identity with the known ALIX V binding sequences in the homologous protein syntenin (encoded by SDCBP1), an interaction that is important for the biogenesis of exosomes^46^. The affinities of the viral SLiMs for their respective protein domains were found to be in the low-to-mid micromolar range (Fig. 3B), which is typical for SLiM-based interactions^14,47^. Viral and endogenous host SLiMs bound with comparable affinities to NEDD4 WW and TSG101 UEV domains. In contrast, the viral ALIX V domain ligand Nsp4_118-142_ (BAV) showed a >300-fold weaker affinity compared to the endogenous ligand derived from syntenin-2 (Fig. 3B). Similarly, the viral CEP55 EABR peptide binders were found to bind the protein with one to two orders of magnitude weaker affinity than the endogenous ligands (Fig. 3G). A higher concentration of the viral ligands would hence be necessary to outcompete the endogenous interactions.

Following up on co-occurring motifs, we noted a close proximity of the ALIX V binding LYPNL motif and the TSG101 UEV binding PTAP motif in Nsp4 of Banna virus (Nsp4_118-142_ (BAV)) (Fig. 3B). We therefore investigated whether the four amino acids separating the two motifs were sufficient to allow simultaneous interaction of both domains with Nsp4_118-142_ (BAV) or if there is competition between the two binding motifs. We challenged a pre-formed complex of ALIX V domain and Nsp4_118-142_ (BAV) peptide with increasing concentrations of TSG101 UEV in the presence of a constant concentration of fluorescein isothiocyanate (FITC)-labeled ALIX V-binding peptide (FITC-gag_493-502_ (HIV1); Fig. 3H). The observed increase in FP signal with increasing concentrations of TSG101 UEV supported a model of mutually exclusive binding of the TSG101 UEV and ALIX V domains to the Nsp4_118-142_ (BAV) peptide. Intriguingly, Banna virus lacks a membrane envelope but could use the ESCRT pathway for non-lytic viral egress or for the formation of double-membrane replication factories as described for the related Bluetongue virus^32^. Non-lytic egress involving hijacking of the ESCRT-pathway components have been described for other non-enveloped viruses such as picornaviruses^48^ and rotaviruses^49^. However, the relevance of the interactions between TSG101 and ALIX with Nsp4 (BAV) remains to be established. Overall, our results for the ESCRT pathway support and complement previous findings.

### RiboVD screening reveals hijacking of clathrin adapters

**Fig. 4:**
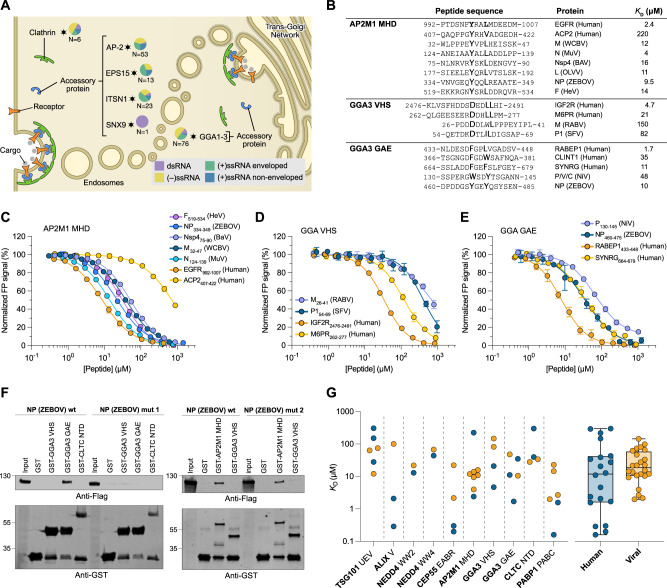
Viral mimicry of distinct trafficking motifs binding to clathrin adapters. **A** Schematic representation of clathrin adapter vesicle coat components for which viral ligands were found: AP2B1 and AP2M1 (collapsed as AP-2), CLTC, EPS15, ITSN1, SNX9, GGA1, GGA2, and GGA3 (collapsed as GGA1-3). Pie charts show the class of viral species hijacking the domain. *N* indicates the number of identified interactions. **B** Overview of affinity data for peptides interacting with clathrin adapter proteins. **C**–**E** FP-monitored affinity measurements of viral and human peptides and host proteins. Data are represented as normalized means ± SD of at least three replicate experiments. **F** Capture of full-length viral proteins by GST-tagged domains as visualized by Western blot. The interaction between NP (ZEBOV) and GGA3 GAE is lost upon motif mutation (Y469A). NP (ZEBOV) also interacts with AP2M1 MHD, but the introduced motif mutations (Y340A/L343A) did not abrogate binding suggesting additional AP2M1-binding SLiMs in NP (ZEBOV). Original blots for these and all subsequent Western blot experiments are provided in Source data files. **G** Overview of all affinity data for viral (orange) and host (blue) peptides generated in this study. In the right panel the combined data is presented as box plot showing mean, interquartile range, and minimum/maximum.

Viruses frequently mimic SLiMs that bind to proteins involved in the endocytic trafficking machinery (Fig. 2; Figure S4). These interactions involve clathrin (discussed in the following section) or its adapters (Fig. 4A). We validated interactions with the Mu homology domain (MHD) of the AP-2 subunits mu (AP2M1 MHD), which is involved in cargo selection and endocytic vesicle formation at the plasma membrane, and with the GAE and the VHS domains of the ADP-ribosylation factor-binding protein GGA3 (GGA3 GAE and GGA3 VHS), involved in cargo recognition and trafficking between the trans-Golgi network and endosomes^50,51^ (Fig. 4B–E). We found that the interactions of the AP2M1 MHD, GGA3 GAE and GGA3 VHS domains (Fig. 4B–D) with viral peptides were in the low-to-mid micromolar range, and that they bound with similar, or lower affinities than the tested endogenous interactions derived from the ELM database (Figure S6; Supplementary data 8). Thus, the results followed a similar trend as observed for the ESCRT pathway ligands described above (Fig. 4B–E, G). We further validated that the nucleoprotein (NP) from Zaire ebolavirus (ZEBOV) has both a _340-_YQQL_−343_ sequence and a _466-_YGEY_−469_ stretch that bind to AP2M1 MHD and GGA3 GAE domain, respectively, with low micromolar affinity (Fig. 4C, E). The interactions between GGA3 VHS and AP2M1 MHD and full-length NP (ZEBOV) were confirmed by glutathione transferase (GST)-pulldown experiments (Fig. 4F; Supplementary data 9). Finally, we confirmed that the interaction between NP (ZEBOV) and GGA3 GAE is motif-dependent, as the interaction was lost upon motif mutation (NP ZEBOV mut 1: Y469A). In contrast, the AP2M1 interaction was retained despite two mutations in the AP2M1-binding motif (NP ZEBOV mut 2: Y340A/L343A). Inspection of the NP sequence revealed six potential AP2M1-binding motifs (YxxΦ), all of which may contribute to binding (Figure S7). These results corroborate previous findings linking the ebolavirus NP to clathrin adapter hijacking^8,52^, and illustrate how a single viral protein can exploit different parts of endocytic trafficking by mimicking different trafficking motifs.

### Eastern equine encephalitis virus Nsp3 interacts with the N-terminal domain of clathrin and blocks receptor trafficking

**Fig. 5:**
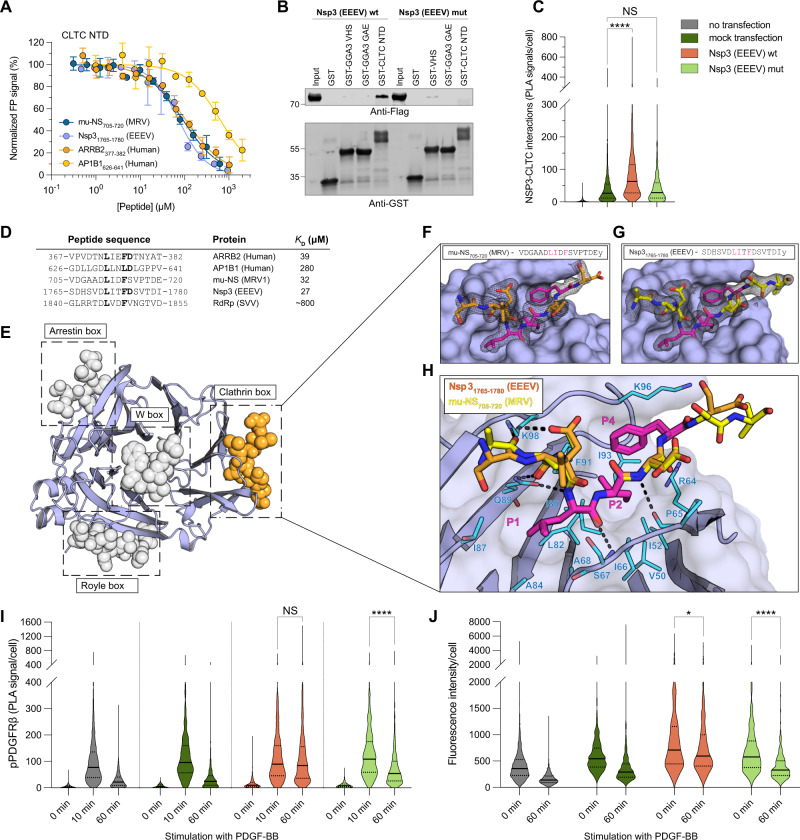
The Nsp3 (EEEV) clathrin box motif is responsible and sufficient for the interaction with clathrin and facilitates the disruption of native cell trafficking. **A** FP-monitored affinity measurements of viral and human peptides binding to CLTC NTD. Data are represented as normalized means ± SD of at least three replicate experiments. **B** Capture of full-length Nsp3 (EEEV) by GST-tagged CLTC NTD visualized by Western blot. **C** The interaction between clathrin and full-length Nsp3 (EEEV) in HEK293 cells probed by proximity ligation assay (PLA). The results show PLA signal per cell (no transfection: *N* = 3270, mock transfection *N* = 1664, Nsp3 (EEEV) wt: *N* = 1075, Nsp3 (EEEV) mut: *N* = 1007) over six biological replicates and are presented as violin plots with indicated median and interquartile range. P (HeV) mut was used as a mock transfection. Significance was determined by Kruskal–Wallis rank sum test with two-sided Dunn’s test and Bonferroni correction as a post hoc test to compare all groups; *p < 0.05, **p < 0.01, ***p < 0.001, and ****p < 0.0001. Source data for all PLA experiments are provided in the Source data file. **D** Alignment of peptides binding to CLTC NTD, with corresponding affinities. **E** Structure of CLTC NTD with four motif-binding sites and bound peptides shown as gray spheres (coordinates for peptides bound to Arrestin, Royle and W boxes were obtained from the PDB entries 1UTC and 5M5T). The peptide bound to the clathrin box is colored orange and represents the binding site of the viral peptides investigated in this study. **F**, **G** Crystal structure of short linear motifs from Nsp3 (EEEV) and mu-NS (MRV1) bound to the clathrin box of CLTC NTD. Panels **F** and **G** show the bound peptides (colored sticks) with the corresponding electron density maps calculated using the final model (black mesh). Conserved motif residues are shown in magenta. **H** Overlay of the two peptides highlighting the conserved binding mode. The CLTC NTD residues engaged in the interactions are shown as blue sticks and hydrogen bonds are highlighted by dotted lines. Coloring is the same as in (**F**) and (**G**). The numbering of the peptide residues starts with P1 being the first position of the consensus recognition motif, while the first residue before the motif is numbered P-1. **I** Activation of pPDGFRβ (phosphorylation of Y751) in the presence of Nsp3(EEEV) in HEK293-PDGFRβ-HA cells probed by PLA. Constructs used are color coded as in (**C**). Results were quantified as in (**C**) (no transfection: *N* = 3029, 3149, and 3134 at 0, 10, and 60 min, respectively; mock transfection: *N* = 872, 923, and 1074 at 0, 10, and 60 min, respectively; Nsp3 (EEEV) wt: *N* = 1203, 1174, and 1365 at 0, 10, and 60 min, respectively, Nsp3 (EEEV) mut: *N* = 1086, 1215, and 1225 at 0, 10, and 60 min, respectively) over three (mock transfection) or six (Nsp3 (EEEV) wt, Nsp3 (EEEV) mut and no transfection) biological replicates. Significance was determined as in (**C**) and asterix denote same statistical significance as in (**C**). Corresponding fluorescence microscopy images for (**C**) and (**I**) are shown in Figure S8 and Figure S11. **J** Quantification of the retention of activated PDGFRβ at the plasma membrane as observed by cell surface fluorescence assay. Constructs used are color coded as in (**C**). Integrated fluorescence intensity was measured over 3 biological replicates (no transfection: *N* = 1940 and 1925 at 0 and 60 min, respectively; mock transfection: *N* = 586 and 1058 at 0 and 60 min, respectively; Nsp3 (EEEV) wt: *N* = 380 and 419 at 0 and 60 min, respectively, Nsp3 (EEEV) mut: *N* = 509 and 453 at 0 and 60 min, respectively). Two-sided Wilcoxon rank sum test with Bonferroni correction for pairwise comparison was used to analyze statistical variance. Asterix denote same statistical significance as in (**C**). Corresponding fluorescence microscopy images are shown in Figure S12.

Next, we focused on viral mimicry of clathrin-binding motifs. The N-terminal domain of clathrin (CLTC NTD) is a β-propeller repeat that binds SLiMs through four different binding sites^53,54^ (Fig. 5E). Our selection revealed three viral peptides containing the classical clathrin box motif (LΦxΦ[DE]): a previously described motif in the mu-NS protein of Reovirus type 1 (MRV1)^55^ together with novel motifs in the Nsp3 protein of the highly pathogenic Eastern equine encephalitis virus (EEEV) and in the RNA-directed RNA polymerase of the Seneca Valley virus. We confirmed the motif-dependent interaction between the Nsp3_1765-1780_ (EEEV) peptide and clathrin by FP affinity measurements and GST pulldown experiments (Fig. 5A, B, D; Figure S6; Supplementary data 9; Nsp3 (EEEV) mut; F1774A/D1775A). We further demonstrated, by an in situ proximity ligation assay (PLA), that the interaction between endogenous clathrin and FLAG-tagged full-length Nsp3 (EEEV) can occur in a cellular setting, mediated by the identified motif (_1771_-LITFD-_1775_) (Fig. 5C; Figure S8).

To further characterize the interactions with clathrin, we solved the structure of CLTC NTD co-crystallized with either Nsp3_1765-1780_ (EEEV) or mu-NS_705-720_ (MRV1) (Fig. 5E–H; Table S1). In both complexes, the structure of the CLTC-NTD was nearly identical, with a root mean square deviation of less than 0.3 Å, and the central eight residues of the peptides well defined in the electron density (Fig. 5F–H). The viral peptides bound exclusively to the hydrophobic clathrin box binding pocket, located between blade one and blade two of the N-terminal β-propeller domain. Structural comparison of the bound viral peptides with an available structure of the host ligand AP2B1 (PDBid: 5M5R; Figure S9)^54^ revealed a similar placement of corresponding residues in the hydrophobic pocket.

The structures supported viral mimicry of the clathrin box motif and a direct competition between viral and human clathrin-binding proteins, which suggested potential interference of Nsp3 (EEEV) with the normal function of clathrin. To explore this competition we used the platelet-derived growth factor receptor β (PDGFRβ) as a model for a receptor tyrosine kinase that is processed via clathrin-mediated endocytosis^56^. After activation by its ligand PDGF-BB, the receptor is phosphorylated at several residues in the cytoplasmic part, internalized primarily via clathrin-mediated endocytosis^57^, and subsequently degraded (Figure S10). We hypothesized that the binding of Nsp3 to clathrin would interfere with clathrin-mediated endocytosis resulting in impaired internalization of activated PDGFRβ. We observed a sharp increase in PLA signal probing for activated PDGFRβ phosphorylated at Tyr751^58^ 10 min after activation with PDGF-BB in all four experimental setups. Consistent with our hypothesis, the signal decreased after 60 min in non-transfected cells, mock-transfected cells, or in cells transfected with a motif-mutant construct Nsp3 (EEEV) mut but persisted in cells transfected with wild-type Nsp3 (EEEV) (Fig. 5I; Figure S11). The clathrin-Nsp3 (EEEV) interaction thus interferes with normal receptor signal attenuation.

To confirm that the activated receptor remained on the cell surface, we performed a cell surface fluorescence assay, which confirmed the presence of PDGFRβ on the plasma membrane 60 min post stimulation, when cells were transfected with Nsp3 (EEEV) wt but not when they were treated with other control constructs (Fig. 5J; Figure S12), further supporting the notion that the Nsp3 (EEEV) interferes with normal clathrin-mediated endocytosis. Importantly, we here used PDGFRβ as a model system, but the results suggest a more general inhibition of clathrin-dependent trafficking. The clathrin-Nsp3 (EEEV) interaction could disrupt surface display of receptors in an analogous manner to HIV1 Nef^59^, or alternatively serve to recruit clathrin to viral replication centers, as previously shown for the clathrin-mu-NS (MRV1) interaction^55^. The exact outcomes of viral clathrin hijacking may warrant further exploration.

### The C-terminal domain of the polyadenylate-binding protein 1 is a target of viral hijacking

**Fig. 6:**
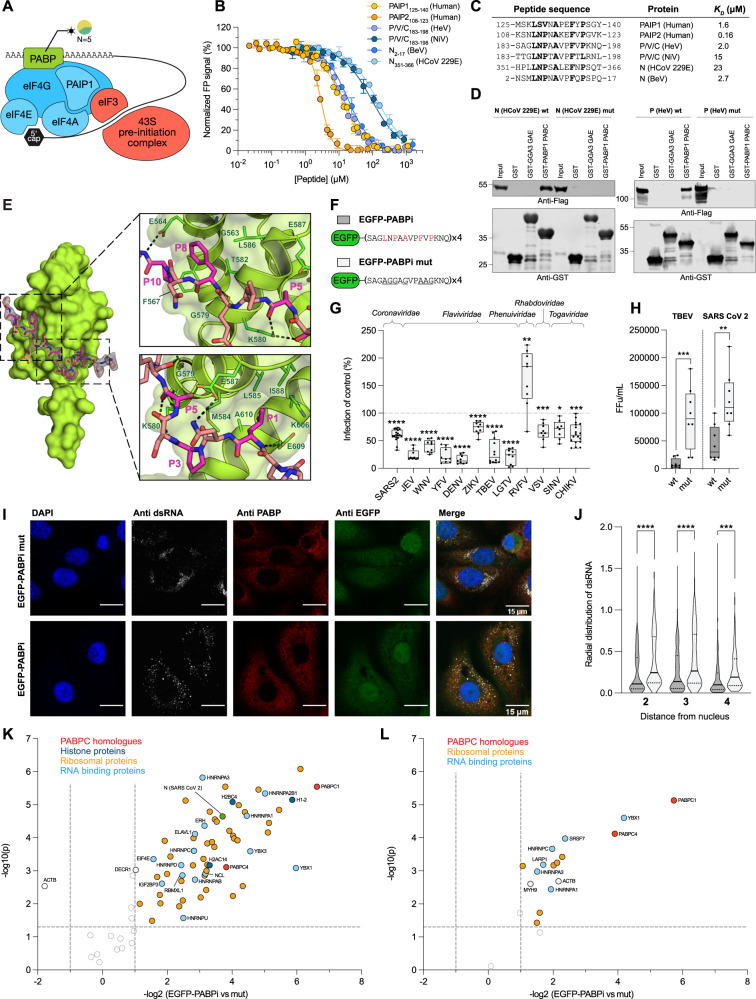
PABP1 is subjected to viral interference and serves as a valid target for broad-spectrum antiviral inhibition. **A** Schematic representation of the closed loop structure that promotes translation initiation and ribosomal subunit recruitment. The components of eIF4F are shown in blue and the pre-initiation complex in red. The pie chart next to PABP1 shows the class of viral species that hijack it. *N* indicates the number of identified interactions. **B** FP-monitored displacement experiments of viral and human peptides and the PABP1 PABC domain. Data are represented as normalized means ± SD of at least three replicate experiments. **C** Alignment of human and viral PABP1 PABC-binding peptides. The residues constituting the recognition motif are shown in bold. **D** Interactions between full-length viral proteins and GST-tagged PABP1 PABC by Western blot. The interaction was lost upon mutation of the PABC interaction motif in viral proteins N (HCoV 229E) mut and P (HeV) mut. **E** Structural model of the PABC domain bound to viral peptide N_351-366_ (HCoV 229E) with corresponding electron density map (black mesh). The two side-panels show the close up of two main binding pockets that facilitate interaction with hydrophobic residues in position P1 and P8 of the peptide. The residues responsible for motif recognition are colored purple. **F** Schematic representation of EGFP-PABPi and the negative control (EGFP-PABPi mut) lentiviral constructs. The specificity determining residues are in red and the residues which were mutated in the negative control construct are underlined. **G** The antiviral effect of EGFP-PABPi against a selection of different RNA viruses compared with EGFP-PABPi mut. Data are cumulative from 3 independent experiments using 3 biologically independent samples (*N* = 9). Statistical analysis was performed using unpaired t test (two-tailed). Data are presented as box plot showing mean, interquartile range and minimum/maximum. Asterix indicates statistical significance, *p < 0.05, **p < 0.01, ***p < 0.001, ****p < 0.0001. Source data for all cell based experiments are provided in the Source data files. **H** Viral titers in the supernatant of cells infected by TBEV and SARS-CoV-2 as determined by focus forming unit (FFU) assay. Data are cumulative from 2 independent experiments using 4 biologically independent samples (*N* = 8) and presented as in (**G**). Statistical analysis was performed as in (**G**). Asterix indicate same statistical significance as in (**G**). **I** Representative confocal microscopy images of VeroB4 cells transduced with EGFP-PABPi or EGFP-PABPi mut and infected with TBEV (multiplicity of infection (MOI) 1) after 24 h. **J** Quantification of the radial coefficient of variation (RadialCV) of dsRNA intensity in the different fractions of the cell. Data are cumulative from 2 independent experiments using 4 biologically independent replicates (EGFP-PABPi: *N* = 158, EGFP-PABPi mut *N* = 185). Statistical analysis was performed as in (**G**). Asterix indicate same statistical significance as in (**G**). **K**, **L** MS analysis of differential expression in lentivirus transduced cells expressing EGFP-PABPi or EGFP-PABPi mut and infected with SARS-CoV-2 (VeroE6; **K**) or TBEV (VeroB4; **L**). MS source data are accessible in the PRIDE database under accession PXD033874.

In order to successfully replicate, viruses need to hijack the host translational machinery^60^. While our screen did not reveal enrichment of interactions with translational machinery proteins, we identified a number of viral peptides that bind to the C terminal domain of polyadenylate-binding protein 1 (PABP1 PABC). PABP1 normally binds to the poly(A) tail of mRNA, stabilizing it and promoting translation initiation (Fig. 6A)^61,62^. PABP1 is commonly degraded by viral proteases to repress translation of endogenous proteins, but can also be subjected to viral hijacking to promote translation of viral proteins^63,64^. Using the PABC domain of PABP1 as a bait, we uncovered interactions with three viral peptides that contain a typical PABP-interaction motif (Fig. 6C). The peptides were found in the non-structural protein P/V/C of the highly pathogenic Hendra virus (HeV; P/V/C_183-198_ (HeV)), and in the nucleoprotein (N) of human coronavirus 229E (N_351-366_ (HCoV 299E)) and Berne virus (N_2-17_ (BeV)). The PABP-binding motif in HeV is also conserved in the closely related Nipah virus (NiV) (P/V/C_183-198_, Fig. 6C). We determined the affinities of PABP1 PABC for four peptides from HeV, NiV, HCoV 229E and BeV (Fig. 6B). The P/V/C_183-198_ (HeV) peptide and the N_2-17_ (BeV) were the highest affinity viral PABC ligands. They bound with similar affinity as the endogenous ligand, PABP-interacting protein 1 (PAIP1_125-140_), but ten-fold weaker than the peptide from the endogenous PABP inhibitor PABP-interacting protein 2 (PAIP2_108-123_) (Fig. 6B, C). The interactions with PABP1 PABC were validated with full-length N (HCoV 299E) and P (HeV) by GST-pulldown (Fig. 6D) and the motif-dependencies of the interactions were validated by mutation of  the seven core residues of the motif (e.g., for P (HeV) residues _186-_LNPAAVPFVP_−195_ were mutated to _186-_AGGAGVPAAG_−195_. For details see Supplementary data 9).

To determine the binding mode of the viral peptides, we attempted to co-crystallize the PABP1 PABC domain with the P/V/C_183-198_ (HeV) or the N_351-366_ (HCoV 299E) peptides. The PABC-N_351-366_ (HCoV 229E) complex crystallized readily, and the structure was solved to 1.93 Å resolution (Table S1). In the complex, the peptide is bound in an extended conformation spanning over two hydrophobic PABC pockets located between helices α2 and α3, as well as α3 and α5, respectively (Fig. 6E). Alignment of the PABP1 PABC-binding peptides showed recurrence of a Leu residue at position 1 and of a hydrophobic residue at position 8 (Fig. 6C), which is a Phe in the N_351-366_ (HCoV 229E) peptide. The structure of PABC-N_351-366_ (HCoV 229E) revealed that the conserved Leu at P1 and Phe at P8 sit in deep hydrophobic pockets which were previously describe to be essential for binding of PAIP2^65^ and PAIP1^66^. A comparison of the binding of N_351-366_ (HCoV 229E) and the human PAIP1 peptide (PDBid 3NTW) revealed a very similar molecular arrangement with a root mean square deviation of <0.4 Å (Figure S9). These results support direct competition between the viral and endogenous PABC ligands.

### The PABC-binding HeV peptide acts as a broad-spectrum inhibitor of viral replication

We reasoned that targeting PABP1 using a PABC-binding peptide could be used to inhibit viral replication of viruses that rely on PABP1 for efficient translation. For example, the Nsp3 protein from Severe acute respiratory syndrome coronavirus 2 (SARS-CoV-2) interacts with the PABP1 ligand PAIP1 to form a ternary complex with PAIP1 and PABP1, which stimulates viral protein translation^64^. We generated a lentiviral construct expressing four copies of the P/V/C_183-198_ (HeV) peptide N-terminally fused to EGFP (EGFP-PABPi) and tested its ability to inhibit infection of a panel of RNA viruses (Fig. 6F, G). EGFP-PABPi reduced the infection level of almost all viruses tested, with the exception of the Rift Valley fever virus (RVFV). The stimulatory effect on RVFV infection by EGFP-PABPi may be related to a previous finding describing the necessity for the RVFV to sequester PABP1 in nuclear speckles for efficient replication^67^. An inhibitory effect of EGFP-PABPi was further demonstrated by low viral titers of the Tick-borne encephalitis virus (TBEV) and SARS-CoV-2 as compared to the control (Fig. 6H). Importantly, EGFP-PABPi did not have any adverse effects on cell proliferation as compared to EGFP-PABPi mut (Figure S13). To analyze how the presence of the EGFP-PABPi affected the viral replication complex in TBEV-infected cells, we detected the viral dsRNA produced within these complexes. We found that the presence of EGFP-PABPi resulted in a more diffuse distribution of the viral replication complexes (Fig. 6I, J, Figure S14). The lower concentration and altered localization of replication complexes could explain the lower viral infectivity, although the exact details of how EGFP-PABPi perturbed the viral infection remain to be elucidated. The results support the notion that targeting the peptide binding pocket of PABC blocks replication of a broad panel of RNA viruses.

Finally, we evaluated the specificity of the EGFP-PABPi peptide for its target in human (HEK293, uninfected) or green monkey cells (TBEV-infected VeroB4 or SARS-COV-2 infected VeroE6) by AP-MS experiments. Consistent with our results, EGFP-PABPi pulled down PABP1, together with its homolog PABP4 in both uninfected HEK293 and virus-infected VeroB4 or VeroE6 cells (Fig. 6K, L; Figure S15; Supplementary data 10). The PABP1/4 proteins were pulled down together with several RNA-binding proteins and with ribosomal proteins, in line with the association of PABP1/4 with the mRNA processing and translation machinery. From cells infected with SARS-CoV-2, EGFP-PABPi additionally pulled down the viral N protein, and its human ligand G3BP1^17^. Overall, the analysis confirmed that the EGFP-PABPi is specific for PABC domain-containing proteins and can successfully be used to attenuate viral replication in a pan-viral manner.

## Discussion

In this study, we present a large-scale pan-viral assessment of how viruses use SLiM-based mimicry to bind host proteins and outcompete endogenous interactors. In total, we found 1712 virus-host PPIs involving 679 viral proteins from 233 viral species, and 97 globular domains from 87 human proteins, yielding an unprecedented, multilayered dataset on virus-host PPIs. We found that all RNA virus families included in this study have SLiMs that can interact with host proteins. Our results fill some of the gaps in host-pathogen interactomes generated by other experimental approaches (e.g., AP-MS and Y2H), with the added value of providing information about the binding motifs with amino acid resolution. The relatively high overlap between the SLiM-focused benchmarking set and the ProP-PD generated data, together with our experimental validations suggests that the results are of high quality. The limited overlap between the current study and other large-scale host-virus interactomics datasets may reflect the biases of different methods. In part, the limited overlap may be due to the composition bias of the reference set, as a large proportion of the reference set is from recent large-scale studies on SARS-CoV-2 host-virus interactomes (Supplementary data 5), and these studies have a relatively low overlap even when comparing interactomes generated using similar methods^68,69^. Our analyses highlight the importance of RiboVD as a resource for the generation of complementary large-scale information on host-virus interactions.

At the highest level, the results give an overview of the processes that are frequently targeted by viruses of different families (Fig. 2). As expected, we found that endocytic transport is a common target of viral hijacking and that different parts of the endocytic machinery are targeted by different viruses with distinct classes and combinations of SLiMs. Closer examination of the data revealed both common strategies of viral hijacking used by unrelated viruses as well as distinct features even among closely related viruses, as demonstrated by the heterogeneous clustering of viruses.

At the molecular level, the results provide exact interaction interfaces in viral proteins. This detailed information can be used to reveal the concerted action of co-occurring motifs in the targeting of human proteins as well as instances of motif competition. We found that adjacent or overlapping SLiMs are common in the IDRs of viral proteomes and likely compete for binding to their host targets (Figs. 2–3). Such mutually exclusive binding could provide temporal control that ensures successful hijacking of vital pathways at the appropriate time in the infection process. Closely located or overlapping WW and TSG101 binding motifs are also found in human proteins such as SIMPLE^70,71^, suggesting that competing motifs interacting with the ESCRT machinery are not unique to viruses but represent a more general regulatory approach.

To gain a deeper understanding of the binding and function of viral SLiMs we analyzed the affinity of 25 virus-host PPIs for ten human protein domains and solved the crystal structures of three complexes. Our results show that the viral ligands bind to the same binding sites as the host ligands and thereby may inhibit host processes, as shown for clathrin-binding Nsp3 (EEEV) (Fig. 5). In contrast to some of the previous literature^2^ proposing that viral SLiMs evolved higher affinities for host targets, our data suggest that viral SLiMs may bind with lower, similar or higher affinities than endogenous SLiMs (Fig. 4G). We find that the affinities of both host-host and viral-host PPIs cover a wide range with no clear pattern as to which has the higher affinity. This is in line with several other studies reporting on similar affinities or even lower affinity of host-virus interactions as compared to the endogenous interaction e.g., with PDZ domains^72–74^ and WW domains^75^. The discrepancy with part of the previous reports may be explained by the fact that some of the viral SLiM-instances previously examined are from proteins expressed early in the viral replication cycle. These early-stage proteins such as E1A (Adenovirus) and E7 (Human papillomavirus) proteins which target Retinoblastoma-associated protein^76,77^, are often present in low concentrations, making high affinity crucial for their function. Conversely the examples of viral SLiM mimicry presented in this study often involved late-stage viral proteins which are expressed at high concentrations. Thus, the key to efficient hijacking by the lower affinity viral ligands may be found in the high local concentration of viral proteins that are generated in virus-infected cells, which is particularly relevant to interactions occurring late during the viral life cycle (e.g. ESCRT pathway). The PABP-binding HeV peptide is an interesting case, as it binds its target with similar affinity to the host ligand PAIP1, a co-activator of translation, but both bind ten-fold weaker than the endogenous PABP inhibitor PAIP2. Thus, the affinities of both viral and host ligands appear to be tuned to the functional role of the interaction (transient binding, or blocking of the target).

Given the omnipresent risk of new emerging viruses, there is an urgent need to systematically map virus-host PPIs and identify targets for development of antiviral agents^3^. We have shown that the PABP1 PABC domain can be targeted to block viral replication in a pan-viral manner. Our results are in line with the previous finding that the endogenous PABP inhibitor PAIP2 restricts cytomegalovirus replication^78^, and demonstrate that the identification and targeting of SLiM-based virus-host PPIs may be a viable strategy for the development of novel antiviral inhibitors. Previous examples of inhibition of viral infection by targeting human proteins include for example targeting of the interaction between the ebolavirus protein VP30 and host protein PP2A-B56^79^, and inhibition of the interaction between N (SARS-CoV-2) and human G3BP1/2^17^. Exploring host proteins as drug targets instead of their viral counterparts is attractive because it has proven more difficult for the virus to evolve resistance to such antiviral agents^18,80^. In addition, the same host proteins or host processes are often targeted by a variety of different viruses, which opens new avenues for the development of broad spectrum antiviral inhibitors, which will contribute towards our preparedness against emerging viral threats^3,81^.

In conclusion, we show that SLiM-based hijacking of host proteins is widespread among RNA viruses. Our data contribute to a better understanding of the molecular details of host cell subversion, and pinpoint novel targets for innovative inhibitor design. Despite the scale of this analysis, we have only started to tap into the host proteins that are targeted by viruses. In the future, we envision studying an even larger collection of bait proteins, including proteins without prior connections to host-virus PPIs. We believe that our study will be valuable to molecular virologists refining the mechanistic understanding of viral infections and that pan-viral data will facilitate the search for novel broad-spectrum inhibitors for use against existing and novel emerging viruses.

## Methods

Reagents and resources are summarized in Table S2.

### Recombinant protein expression and purification

Proteins (Supplementary data 2) were expressed in *E. coli* BL21(DE3) as GST-tagged proteins in 2YT growth media (16 mg/mL peptone, 10 mg/mL yeast extract, 5 mg/mL NaCl) supplemented with appropriate antibiotics (50 µg/mL kanamycin (Kan) for pETM33 constructs and 100 µg/mL ampicillin (Amp) for pHH1003 constructs) at 37 °C. After reaching an OD_600_ of 0.6, protein expression was induced with 1 mM isopropyl β-D-1-thiogalactopyranoside (IPTG). Proteins were expressed either for 4 h at 30 °C or overnight at 18 °C. Bacterial cultures were harvested by centrifugation (4500 × *g*, 10 min) at 4 °C and resuspended in lysis buffer A (PBS supplemented with 1% Triton, 10 µg/mL DNase I, 5 mM MgCl_2_, 10 µg/mL of lysozyme, and cOmplete™ EDTA-free Protease Inhibitor Cocktail (Hoffman-La Roche) when the protein was used for phage display selections, or in lysis buffer B (50 mM Tris/HCl pH 7.8, 300 mM NaCl, 10 µg/mL DNase I and RNase, 4 mM MgCl_2_, 2 mM CaCl_2_ and cOmplete EDTA-free Protease Inhibitor Cocktail) when the protein was used for FP affinity determination experiments. Cells were lysed either with two cycles of 20 s sonication with 2 s pulses, or with a cell disruptor apparatus at 1.7 kBar. The lysate was clarified by centrifugation (20,000 × *g*, 40 min) and the supernatant was filtered through a 0.2 µm sterile PES filter, transferred to Pierce Glutathione Agarose and purified according to the manufacturer’s protocol. For proteins used in FP experiments additional purification steps were performed. After elution, the His/GST tag was enzymatically cleaved with either Thrombin or PreScission protease overnight at 4 °C. The sample was then applied to a nickel Sepharose excel resin and the protein of interest was collected in the unbound fraction. Protein samples were transferred into 50 mM potassium phosphate buffer pH 7.5 using HiPrep 26/10 desalting column. All protein samples were analyzed by SDS-PAGE gel electrophoresis and the protein concentration was determined based on absorbance and extinction coefficients calculated from the amino acid sequence. Correct protein identity was confirmed by matrix-assisted laser desorption/ionization time-of-flight mass spectrometry (MALDI-TOF/MS).

### Phage display and analysis of NGS results

The RiboVD phage library displays the intracellular IDRs of mammalian and avian RNA viruses (Riboviria; taxonomic identifier: 2559587) tiled by 16 amino acids overlapping peptides (Supplementary data 1)^17^. The library design is available on-line (http://slim.icr.ac.uk/phage_libraries/rna_viruses/species.html). Briefly, transmembrane and extracellular regions (as defined by UniProt) were removed. IDRs were defined by using surface accessibility scores from structures of the protein or from homology models and from disorder predictions using IUPred (cut-off 0.4)^14^.

The library was used in triplicate phage selections against 139 His-GST/MBP tagged bait protein domains (Supplementary data 2). Proteins (10 µg in 100 µL PBS) were immobilized in 96-well Flat-bottom Immunosorp MaxiSorp plates for 18 h at 4 ˚C. Wells were blocked with 200 µL BSA (0.5% in PBS) and washed four times with 200 µL PT (PBS + 0.05% (v/v) Tween 20) before adding the phage library (10^11^ phage in 100 µL PBS per well), first to the GST-coated wells (1 h) to remove non-specific binders, and then to the bait protein-coated plates (2 h). Unbound phages were removed and the bound phages were eluted (100 µL log phase *E. coli* OmniMAX, 30 min, 37 ˚C). M13 helper phages were added (10^9^ M13KO7 helper phages per well, 45 min at 37 ˚C) before transferring the bacteria to 1 mL 2xYT supplemented with 100 µg carbenicillin (Carb), 30 µg Kan and 0.3 mM IPTG. Bacteria were grown at 37 ˚C for 18 h, before harvesting the phages (2000 × *g* for 10 min). The phage supernatants were pH adjusted (using 1/10 volume 10x PBS) and used as in-phage for the next round of selection.

The peptide-coding regions of the naive RiboVD library and the binding-enriched phage pools (5 µL) were PCR-amplified and barcoded using Phusion High-Fidelity polymerase (Thermo Scientific) for 22 cycles. PCR products were confirmed by 2% agarose gel electrophoresis stained with GelRed using a 50 bp marker (BioRad). PCR products were normalized using Mag-bind Total Pure NGS, pooled and purified from a 2% agarose gel (QIAquick Gel extraction Kit), and analyzed using Illumina MiSeq v3 (1 × 150 bp read setup, 20% PhiX). Results were processed using in-house Python scripts. Reads were demultiplexed, adapter and barcode regions were trimmed, and sequences were translated into peptide sequences. Peptides were annotated using PepTools^14^. The state of viral protein annotation in UniProt is ever-changing, and multiple strains of the same viral species sometimes have multiple entries for the same (or very similar) proteins and polyproteins. Our annotations with PepTools takes this situation into account. When counting the number of interactions, we opted to collapse the viral proteins based on a combined IDs that include their names (not accessions), chain names (not chain IDs), and species (at the species level, not strain).

Confidence levels were assigned based on four different criteria: occurrence in replicate selections, identification of overlapping peptide sequences, high counts, occurrence of sequences matching consensus motifs determined from the generated dataset, or a priori defined consensus motifs for the bait proteins^4,14^. For a stringent analysis we focused on the medium/high confidence peptides that fulfill at least two of these criteria, where the fulfillment of two or three of the criteria results in a peptide being considered medium confidence. For a peptide to be defined as high confidence it must fulfill all four criteria. In addition, we apply a specificity filtering with a cut-off value of 0.2. The specificity of a peptide for a bait domain was calculated as the proportion of the total NGS read counts of a given peptide for a bait domain in comparison to the combined NGS reads for the peptide for all baits screened. The specificity values were calculated on the domain level and not the bait level by grouping the baits using their Pfam domain family (e.g. the specificity score of a peptide for the NEDD4 WW domain is the same the specificity score of all other WW domains).

The quality metrics were benchmarked previously^14^ and here evaluated against the RiboVD motif benchmarking set (Figure S2). For each bait, the RiboVD selected peptides that overlap with the validated motif instances for that bait in the RiboVD motif benchmarking set were compared to all other selected peptides. Six metrics were compared: (i) confidence level, (ii) replicated peptides (the number of replicates that the peptides are observed in), (iii) overlapping peptides (the number of distinct peptides overlapping the motif across all replicates), (iv) specificity determinant match (the SLiMFinder-derived PSSM match *P*-value), (v) normalized peptide count (the mean normalized peptide count for the peptide across the NGS counts of the replicates), and (vi) the specificity score.

### Enrichment analysis of co-occurring SLiMs

To calculate the p-value scores for the enrichment of viral co-occurring SLiMs targeting human bait-bait pairs, first a dataset for peptide sampling was created from all peptides with a confidence level of 2 or greater in the complete screening results. For each human bait-bait pair, peptide samples of the same size as the number of peptides of confidence level 2 or greater for each bait were created from the peptide dataset and the number of viral proteins shared between the bait pair was determined (the intersection of the 2 randomized samples). This step was repeated 1,000,000 times to create a distribution of shared viral protein counts for each human bait-bait pair. This shared protein count was then used to calculate the probability of the observed overlap (Supplementary data 6). Only human bait-bait pairs with different domain types (as defined by their Pfam identifiers) were used for this analysis.

### Viral network generation and analysis

The Human PPI network was extracted from IntAct (version: 4.2.17, last update May 2021)^25^. We also included kinase-kinase interactions and kinase-substrate interactions from PhosphoSitePlus^25^ (version 6.5.9.3, last update May 2021), OmniPath^82^ (last release May 2021) and SIGNOR 2.0^83^ (last release May 2021). Only proteins annotated in Swiss-Prot^84^ and those annotated with at least one GO term^85^ were kept. The resulting protein interaction network (PIN) comprises 16,407 nodes and 238,035 edges. Edge weights are modeled according to the Topological Clustering Semantic Similarity^86^ and calculated using the Semantic Measure Library^87^. In addition, to determine the significance associated with each node, we generated 1000 random networks employing the configuration model available in the python igraph library (http://igraph.org) updating the edge weight accordingly. Each network is Laplacian-normalized to correct for the hub bias. In the formula:

1 $${w}_{{ij}}=\frac{{w}_{{ij}}}{\sqrt{{d}_{i}{d}_{j}}}$$

where *w*_*ij*_ indicates the edge weight (i.e., semantic similarity) and *d*_*i*_ and *d*_*j*_ represent the weighted degree of node *I* and node *j*.

The Random walk with restart (RWR) algorithm (though the personalized PageRank function available in http://igraph.org was used to simulate the propagation of viral infection into the PIN. The human proteins targeted by the virus were selected as seed nodes for the RWR procedure selecting a damping factor equal to 0.7. The RWR algorithm was also executed on the 1000 random networks employing the same seed nodes and restart probability. This allows us to estimate the empirical *p*-value for each protein in the PIN as the percent of random score that exceeded the real score (excluding the seed genes), that is:

2 $$p-{{{{{{\rm{value}}}}}}}=1-\frac{\left\{I{{{{{\rm{|}}}}}}{{{{{{{\rm{RWR}}}}}}}}_{{{{{{{\rm{empirical}}}}}}}} > {{{{{{{\rm{RWR}}}}}}}}_{{{{{{{\rm{random}}}}}}}}\right\}}{1000}$$

Where *I* is the indicator function, RWR_empirical_ and RWR_random_ refer to the RWR score assigned to the empirical PIN and the random networks, respectively. Only nodes with a *p*-value < 0.01 are considered significant. In total 575 target networks, one for each virus, from 26 different viral families are extracted. Each target network is represented by a vector comprising the significant RWR scores associated with the proteins belonging to the target network. To identify the common biological processes subjected to the viral interference, the human nodes in the networks that are significantly affected by viral infection are selected. To do so, for each protein in the networks, we defined the average RWR family specific score as:

3 $${{{{{\mathrm{Average}}}}}}\; {{{{{\mathrm{RWR}}}}}}=\frac{{\sum }_{i=1}^{n}{{{{{{\mathrm{RWR}}}}}}}_{{{{{{\mathrm{score}}}}}}}}{{{{{{\mathrm{\#}}}}}}\,{{{{{\mathrm{of}}}}}}\; {{{{{\mathrm{viruses}}}}}}\,{{{{{\mathrm{within}}}}}}\,{{{{{\mathrm{the}}}}}}\,{{{{{\mathrm{family}}}}}}}$$

Representing the average RWR score assigned to each significant protein belonging to the respective family. To assess which average RWR score is significant, we calculated the upper-tailed Z-Score test, employing as background distribution the random walk scores of those nodes that didn’t pass the significance threshold (i.e., *p*-value > 0.01). Proteins with a score in at least 8 viral families and with a Z-Score >2.32 (corresponding to a *p*-value < 0.01) were selected. This set constitutes the foreground for the enrichment analysis against GO using the human proteome as background. The background comprised all proteins in the protein interaction network that we derived by performing the network propagation with the same parameters, using all the baits as seeds rather than only the hits for the respective viral peptides. We used g:Profiler^88^ to perform enrichment analysis (Supplementary data 7), focusing on the biological process domain. Then, we employed Enrichment Map^89^ and Cytoscape^90^ to visualize the GO biological process map.

### Cluster network families

Firstly, for each of the 575 viral signatures, we performed an enrichment analysis against Reactome^91^ using all levels of the pathway hierarchy. Fisher’s exact test^92^ based on the hypergeometric distribution is used to determine the overrepresented terms and the Bonferroni correction^93^ is applied to correct for multiple comparisons. To extract the 26 family signatures, we summed the corresponding RWR scores of the proteins in the viral signature vectors appertaining to their respective family. After this procedure, a matrix A of 26 × 4275 elements is obtained, where each row corresponds to a family signature and each column represents the sum of the RWR scores for each protein within their respective family. A value equal to 0 is assigned if the protein was not significant in any of the viral signatures within that family. Since the distribution of the viruses inside each viral family was different, we normalized the matrix using the quantile normalization from the *scikit-learn* package^94^ matrix using the quantile normalization from the scikit-learn package. Next, since the normalized matrix was positive, we applied the standard non-negative matrix factorization (NMF) from the *nimfa* library with default parameters (latent factor a part)^95^ to identify groups of viral families targeting similar human pathways. A critical step in NMF was to select the right number of latent factors. For this aim, we ran the NMF algorithm 1000 times employing the initialization algorithm to obtain a stable consensus clustering^96^. In each run, we calculated the cophenetic correlation coefficient. We selected 5 latent factors as evident from the violin plot (Figure S4), since increasing the number of latent factors slightly increased the cophenetic correlation coefficient. Hence the normalized matrix A was decomposed into:

4 $$A\sim {WH}$$

The maximum value on each row of the coefficient matrix H represents the strongest membership of the family with the latent component and consequently a cluster. We calculated the mean of the relative frequency of a Reactome pathway within the family inside each cluster:

5 $${{{{{\mathrm{Relative}}}}}}\,{{{{{\mathrm{frequency}}}}}}={{{{{\mathrm{mean}}}}}}\left(\frac{{{{{{\mathrm{\#}}}}}}{{{{{\mathrm{Reactome}}}}}}\,{{{{{\mathrm{pathway}}}}}}\,{{{{{\mathrm{enriched}}}}}}\,{{{{{\mathrm{within}}}}}}\,{{{{{\mathrm{the}}}}}}\,{{{{{\mathrm{family}}}}}}}{{{{{{\rm{\#}}}}}}{{{{{\mathrm{viruses}}}}}}\,{{{{{\mathrm{in}}}}}}\,{{{{{\mathrm{the}}}}}}\,{{{{{\mathrm{family}}}}}}}\right)$$

and the absolute frequency of that pathways inside the cluster:

6 $${{{{{\mathrm{Global}}}}}}\,{{{{{\mathrm{frequency}}}}}}=\frac{{{{{{\mathrm{\#}}}}}}{{{{{\mathrm{Reactome}}}}}}\,{{{{{\mathrm{pathways}}}}}}\,{{{{{\mathrm{in}}}}}}\,{{{{{\mathrm{the}}}}}}\,{{{{{\mathrm{cluster}}}}}}}{{{{{{\mathrm{\#}}}}}}{{{{{\mathrm{of}}}}}}\,{{{{{\mathrm{viruses}}}}}}\,{{{{{\mathrm{in}}}}}}\,{{{{{\mathrm{the}}}}}}\,{{{{{\mathrm{cluster}}}}}}}$$

To consider a Reactome pathway representative of each cluster both scores must be greater than 0.2 (see Fig. 2B).

To compare the vesicle-mediated transport networks, we extracted all the enriched proteins involved in the endocytosis pathway for cluster 4 and 5, respectively, for each of the families involved and analyzed them using Cytoscape.

### Affinity measurements

Affinity measurements were performed in 50 mM potassium phosphate pH 7.5, or 50 mM potassium phosphate pH 7.5, 1 mM TCEP. Experimental setup and conditions were identical for all domains unless stated otherwise. The affinity between the protein domains and their respective FITC-labeled peptides was determined with saturation binding experiments (Figure S6). A 1:1 dilution series with increasing concentration of protein of interest was performed containing a fixed concentration of FITC-labeled peptide (ranging from 5 to 10 nM depending on the protein under investigation) in black, non-binding surface, flat bottom 96-well plates. Measurements were performed on a SpectraMax iD5 plate reader at room temperature and at excitation/emission wavelengths of 485/535 nm. The G-factor was set accordingly so that the wells containing only the FITC-labeled peptide showed a fluorescence polarization value between 10–40 mP (corresponding to *B*_bottom_). Saturation binding curves were analyzed by GraphPad Prism and fitted to the equation:

7 $$Y={B}_{{{{{{{\rm{bottom}}}}}}}}+\frac{X\times {B}_{{{{{{{\rm{amp}}}}}}}}}{{K}_{D}+X}$$

where *B*_bottom_ is the fluorescence polarization value of FITC-labeled peptide in absence of protein, *B*_amp_ is the amplitude of fluorescence polarization signal (*B*_top_ − *B*_bottom_), X is the concentration of free protein (equal to total protein since [protein]»[FITC-peptide]), *K*_D_ is the equilibrium dissociation constant and Y is the fluorescence polarization signal.

To determine affinities between proteins and non-labeled peptides a competition assay was performed. The non-labeled peptide was added at increasing concentrations to a fixed concentration of FITC-labeled peptide (5–10 nM final concentration, depending on the protein) and protein of interest. Fixed concentrations of proteins in displacement experiments were as follows to achieve approximately 60% saturation of the complex between protein and labeled peptide: ALIX V: 4–6 µM, TSG101 UEV: 8 µM, NEDD4 WW2: 30 µM, NEDD4 WW4: 30 µM, CEP55 EABR: 1 µM, GGA3 VHS: 15–17 µM, GGA3 ear: 4 µM, CLTC NTD: 30 µM, AP2M1: 0.9–1.65 µM and PABP1 C: 1.76–2 µM. FP values from the competition assay were fitted (GraphPad Prism) to a sigmoidal dose-response equation

8 $$Y={B}_{{{{{{{\rm{bottom}}}}}}}}+({B}_{{{{{{{\rm{amp}}}}}}}})/(1+{10}^{((\log {{{{{{\rm{IC}}}}}}}50-X)\times {{{{{{\rm{nH}}}}}}})})$$

where *Y* is the fluorescence polarization signal, *B*_bottom_ is the FP value of FITC-labeled peptide in absence of protein, *B*_amp_ is the amplitude of FP signal (*B*_top_ − *B*_bottom_), IC50 is non-labeled peptide concentration required for 50% apparent inhibition, *X* is the logarithmic value of non-labeled peptide concentration and nH is the Hill coefficient. The resulting IC50 values obtained from the displacement experiment were converted to *K*_D_ values as previously described^97^. All *K*_D_ values were calculated on the raw fluorescence polarization data. Normalization was employed to facilitate easier visualization. All saturation and competition experiments were performed at least in three technical replicates.

### Crystallization

The CLTC NTD was co-crystallized with two viral peptides that were also used in affinity measurement studies namely Nsp3_1765-1780_ (EEEV) and mu-NS_705-720_ (MRV1), by vapor diffusion method (MRC 2 Well Crystallization Plate in UVXPO; Hampton research). CLTC NTD concentrated to 18 mg/ml in 50 mM Tris-Cl (pH-7.7), 200 mM NaCl, 4 mM DTT was mixed with peptides dissolved in the same buffer at 10 mg/ml at a protein:peptide ratio of 1:2 and stored at −20 °C until crystallization plate setup. Initially, the crystallization was attempted by using reported crystallization conditions (50 mM Tris-Cl pH-7.5 and 3% PEG 6000)^98^. The crystal growth was optimized by varying the pH of Tris (pH 7.0–8.5) and concentration of PEG 6000 (20–30%). For both peptides the plate-like crystals appeared in several drops within 2 days. Microseed stocks were prepared for each of the CLTC NTD-peptide complexes from the crushed crystals harvested from a single drop, diluted 1:100 with the respective mother liquors. These stocks were used to screen the conditions of the Morpheus crystallization screen^99^ in a sitting-drop setup. For each complex, single crystals appeared under several conditions. The best diffracting CLTC NTD-Nsp3_1765-1780_ (EEEV) crystals were grown using 30% PEG 550 MME/PEG 20 K and 0.1 M NPS buffer system pH 6.5 (containing NaNO_3_, Na_2_HPO_4_, and (NH_4_)_2_SO_4_) as reservoir solution. The best CLTC NTD-mu-NS_705-720_ (MRV1) crystals were obtained with 30% PEG 550 MME/PEG 20 K, 0.12 M monosaccharides (D-Glucose, D-Mannose, D-Galactose, L-Fucose, D-Xylose, N-Acetyl-D-Glucosamine), and 0.1 M sodium HEPES/MOPS pH 7.5. Crystals were cryo-cooled in liquid nitrogen without additional cryoprotectant.

PAPB1 PABC domain was concentrated to 20 mg/ml in 50 mM Tris (pH-7.5), 150 mM NaCl, 1 mM DTT and incubated with the N_351-366_ (HCoV229E) peptide at 1:1.5 molar ratio. The ammonium sulfate screen (AmSO_4_ suit, Hampton Research) was used to identify the initial crystallization conditions at 22 °C. The crystallographic data were collected from crystals grown using a reservoir solution of 0.1 M sodium MES pH 6.5, 1.8 M ammonium sulfate. Crystals were briefly soaked in mother liquor containing 20% glycerol prior to cryo-cooling in liquid nitrogen.

### X-ray data collection, structure determination, and refinement

For the two peptide complexes of CLTC, crystallographic data was collected at 100 K at the beamline I04 of the Diamond Light Source (Didcot, UK) and processed on site using either Fastdp or Xia2^100^. The structures were solved by molecular replacement using Phaser^101^ and PDB entry 1C9I as search model^102^. The PABPC1 PABC-HCoV 229E data were collected at BioMAX, MAX IV^103^ (Lund, Sweden), and processed at the beamline using the autoproc pipeline^104^. The structure was solved by a molecular replacement method using Phaser and PDB entry 3KUJ^65^ as the search model. All three structures were refined with phenix.refine and Refmac5 of the Phenix^105^ and CCP4 program suites^106^, respectively. Manual model building was done in *Coot*^107^. The final structures showed good geometry as analyzed by Molprobity^108^. The data collection and refinement statistics are given in Table S1.

### Cells and viruses

Human embryonic kidney 293 cells (HEK293) (Sigma), HEK293T (TakaraBio), and African green monkey kidney E6 cells (VeroE6) cells (ATCC, CRL-1586) were cultured in Dulbecco’s modified Eagle’s medium (DMEM)(Gibco) supplemented with 10% (v/v) fetal bovine serum (FBS) (HyClone) and 100 units/ml penicillin G with 100 μg/ml streptomycin solution (PEST) (Gibco) at 37 °C, 5% CO_2_, humidified chamber unless otherwise specified. The African green monkey kidney B4 cells (VeroB4) cells were cultured in 199/EBSS medium (HyClone) supplemented with 10% (v/v) FBS, and PEST. For PLA, HEK293, and HEK293 overexpressing HA-tagged human PDGFRβ (HEK293-PDGFRβ-HA, a kind gift from Frank Böhmer^109,110^) were cultured in DMEM and Nutrient Mixture F-12 (1:1) (Gibco) supplemented with 10% (v/v) FBS (Gibco) and PEST.

SARS-CoV-2 (SARS-CoV-2/01/human2020/SWE accession no/GeneBank no MT093571.1, provided by the Public Health Agency of Sweden), was grown in VeroE6 cells and used at passage number 4. Japanese encephalitis virus (JEV) (Nakayama strain), West Nile virus (WNV) (WNV_0304h_ISR00), yellow fever virus (YFV) (Asibi), and dengue virus (DENV) (serotype-2; PNG/New Guinea C) were kind gifts from S. Vene, the Public Health Agency of Sweden and were grown in VeroB4 cells. TBEV (Torö−2003^111^, Langat virus (LGTV) (TP21, kind gift from Gerhard Dobler Bundeswehr Institute of Microbiology, Munich, Germany), ZIKV (MR766, kind gift from Gerhard Dobler Bundeswehr Institute of Microbiology, Munich, Germany), RVFV^112^, vesicular stomatitis virus (VSV) (kind gift of Friedemann Weber, University of Freiburg), Sindbis virus (SINV) (Lovanger, KF737350, kind gift from Olivia Wesula Luande) and chikungunya virus (CHIKV) (CHIKV LR2006OPY1, kind gift from Magnus Evander) were grown in VeroB4 cells.

### GST-pull down assay

The GST pulldown assay was performed using a previously established protocol^113^. Whole-cell lysates were obtained by transfecting HEK293T cells cultured on 100 mm culture plates with plasmids expressing C-terminal Flag-tagged NP (ZEBOV) wt, NP (ZEBOV) mut 1, NP (ZEBOV) mut 2, Nsp3 (EEEV) wt, Nsp3 (EEEV) mut, N (HCoV 229E) wt, N (HCoV 229E) mut, P (HeV) wt and P(HeV) mut proteins. 48 h post transfection, the cells were harvested, washed with 1 X PBS and lysed in GST-lysis buffer containing 25 mM Hepes-KOH (pH 7.4), 12.5 mM MgCl_2_, 100 mM KCl, 0.1 mM EDTA, 10% glycerol, 0.1% NP-40, supplemented with protease inhibitor for 30 min on ice. The cell lysates were freeze-thawed three times and the supernatant was collected by centrifugation at maximum speed for 15 min. The cell lysates were incubated with GST-tagged proteins for 1 h, at room temperature with end-over-end mixing. The beads were washed with the GST-lysis buffer and the bound proteins were separated by SDS-PAGE and analyzed by western blotting. For western blotting, the SDS-PAGE separated proteins were transferred onto nitrocellulose membrane (Amersham, Protran) for 2 h, 200 mA at 4 °C. The membrane was blocked in Odyssey blocking buffer (LI-COR) for 1 h at room temperature and incubated in primary antibodies anti-mouse Flag (Sigma, M2, F1804), anti-rabbit GST (Santa Cruz, sc-33614), overnight at 4 °C. The membrane was washed three times in PBS-T (PBS + 0.1% Tween 20) before incubation with fluorescent secondary antibodies (IRDye®, LI-COR) against anti-mouse or anti-rabbit for 30 min at room temperature. The membrane was washed three times in PBS-T and scanned using Odyssey scanner (LI-COR).

### Proximity ligation assays

HEK293 cells were seeded in 8-well Nunc Lab-Tek II chamber slides (0.7 cm^2^, Sigma) at a density of 70.000 cells/cm^2^. After 40 h, the cells were transfected with plasmids expressing C-terminal Flag-tagged Nsp3 (EEEV) wt, Nsp3 (EEEV) mut, P (HeV) mut proteins (Supplementary data 9) or not transfected. Growth medium was replaced with Opti-mem (ThermoFisher) and the cells transfected with 100 ng Plasmid DNA per well using Lipofectamine 3000 (ThermoFisher) as described by the manufacturer. After 6 h of incubation, the medium was replaced with the growth medium and grown overnight. On ice, cells were washed in ice-cold PBS, then fixated in ice cold formalin solution (3.7% paraformaldehyde plus 1% methanol in PBS) for 15 min before washing in PBS 3 times for 5 min. The slides were dried and the wells encircled with an ImmEdge hydrophobic barrier pen (Vector Laboratories). The slides were rehydrated in TBS and the cells permeabilized in TBS plus 0.2% Triton X-100 for 10 min. In a moisture chamber, the slides were blocked in blocking buffer consisting of Odyssey Intercept (TBS) Blocking Buffer (Licor) plus TBS in a 1:1 ratio for 1 h at 37 °C, before incubation overnight at 4 °C with primary antibodies goat-anti-FLAGtag (ab1257, Abcam) (1:1000) and mouse-anti-clathrin (ab2731, Abcam) (1:200) diluted in blocking buffer. The slides were washed 3 times 10 min in TBS plus 0,05% Tween-20 before incubation with Duolink secondary probes (Olink) compatible with host species of the primary antibodies. The slides were incubated for 1 h at 37 °C with Duolink PLA probe anti-Mouse PLUS and Duolink PLA probe anti-Goat MINUS diluted in blocking buffer to a concentration of 1x. The slides were washed 3 times for 10 min in TBS plus 0.05% Tween-20 and incubated with 1x Duolink Ligation solution and 1 U/μL T4 DNA ligase (Thermo Fisher) for 30 min at 37 °C. The slides were washed 3 times for 10 min in TBS and incubated with 1x Duolink Amplification Red solution and 0.125 U/μL Phi 29 polymerase (Montserate) and washed again 3 times for 10 min in TBS. To visualize transfected cells, the slides were incubated with Donkey anti-goat Alexa Flour Plus 647 (A32849, Thermo Fisher) and Hoechst 33342 for 1 h at 37 °C. The slides were washed again 3 times for 10 min in TBS plus 0.05% Tween-20, then briefly washed in TBS and mounted with Slowfade Gold antifade mounting reagent (S36936, Thermo Scientific).

PLA experiments with PDGFRβ were performed using HEK293 overexpressing HA-tagged human PDGFRβ (HEK293-PDGFRβ-HA). The PLA experiments were performed as described above, except after transfection the cells were starved overnight in starvation medium (DMEM/F-12, 0.2%FBS) and then stimulated with 50 ng/ml PDGF-BB (Peprotech) in starvation medium for 0, 10, and 60 min at 37 °C before fixation. Primary antibodies used were rabbit-anti-PDGFRβ (#3169, Cell Signaling Technology) (1:100) and mouse-anti-PDGFRβ-pY751 (#3166, Cell Signaling Technology) (1:200), and Duolink PLA probes were anti-Mouse PLUS and Duolink PLA probe anti-Rabbit MINUS. To visualize transfected cells, the slides were incubated with FLAG-tag antibody (1:1000) for 1 h at room temperature, washed 3 times for 10 min in TBS plus 0,05% Tween-20, and subsequently incubated with secondary antibody Donkey anti-goat Alexa Fluor Plus 647 (A32849, Thermo Fisher) diluted 1:500 and 10 μg/mL Hoechst 33342 in blocking buffer for 1 h at 37 °C. The slides were washed 3 times for 10 min in TBS plus 0.05% Tween-20 and mounted as previously described.

Slides were imaged using a Zeiss Imager Z2 controlled by Zen 2 (blue edition) software. The microscope was equipped with a Hamamatsu C11440 camera, a 40x/1.4 oil objective, filter cube sets 31, 43 HE, 49, and 50 from Zeiss, and a HXP 120 V light source set to 90% for all channels imaged. 3 images per condition for each experiment were acquired as z-stacks of 11 slices 0.5 μm apart. The images shown are the maximum intensity projection of the z-stack and have been adjusted for brightness and contrast for visualization purposes.

Image analysis and quantification of PLA signal was performed using CellProfiler software version 3.0.0 and v.4.2.4, made available by the Broad Institute Imaging Platform^114^. Image analysis was performed on the maximum intensity projection of the z-stack of original images. Segmentation of the cells was performed based on the image resulting from the Hoechst channel using first the IdentifyPrimaryObjects module for segmentation of nuclei based on a global three-class Otsu threshold method using intensity to distinguish and draw dividing lines between clumped objects, followed by the IdentifySecondaryObjects module to segment cells using the Distance-N function with a fixed maximum distance from the nucleus to cell border. The PLA signal was evaluated as PLA rolling circle amplification product (RCP) per cell. The image from the TexasRed channel was first filtered with the help of the EnhanceOrSuppress module to enhance the feature type “speckles” and remove background. The filtered image from the TexasRed channel was then used as input for segmentation of RCPs, based on manual thresholding using the IdentifyPrimaryObjects module. RCPs were then related to the cells via the RelateObjects module. Integrated intensity per cell was measured using the MeasureObjectIntensity module for the channel imaging the FLAGtag. Finally, all intensity measures and RCPs per cell were exported to an Excel spreadsheet. To distinguish data from transfected and non-transfected cells, a cutoff intensity for transfected cells was set corresponding to the highest integrated intensity per cell of the FLAGtag containing channel for non-transfected cells.

### Cell surface fluorescence assay

HEK293-PDGFRβ-HA cells were seeded, transfected, and stimulated for 0 or 60 min as described for PLA experiments. On ice, the cells were washed in ice-cold PBS and incubated with a primary antibody targeting the extracellular part of PDGFRβ, 5 μg/ml goat-anti-PDGFRβ (AF385, RnD Systems) in PBS for 1 h. The cells were washed 3 times for 10 min in PBS before fixation, permeabilization, and blocking was performed as described for PLA experiments. The cells were incubated with rabbit-anti-FLAG (1:800) (#14793S, Cell Signaling Technology) diluted in blocking buffer overnight at 4 °C, washed 3 times 10 min in TBS plus 0.05% Tween-20, and subsequently incubated with secondary antibodies Donkey anti-rabbit Alexa Fluor Plus 555 (A32794 ThermoFischer) and Donkey anti-goat Alexa Fluor Plus 647 (A32849, ThermoFischer) diluted 1:500 and 10 μg/mL Hoechst 33342 Solution (Thermo Scientific) (1:1000) in blocking buffer. The slides were washed 3 times for 10 min in TBS plus 0.05% Tween-20 and mounted and observed under microscope as described for PLA experiments. Images were analyzed with CellProfiler, using the same pipeline for segmentation and distinguishing between transfected and untransfected cells as described for PLA experiments. Fluorescence intensity was measured as integrated intensity per cell for the channel imaging PDGFRβ using the MeasureObjectIntensity module.

### Lentivirus plasmids and production

Lentiviruses were produced by transfection of HEK293T cells in 100 mm plates^17^. To produce lentiviruses, pLJM1-EGFP (David Sabatini lab, Addgene plasmid #19319^115^), psPAX2 (Didier Trono lab, Addgene plasmid #12260), and pMD2.G (Didier Trono lab, Addgene plasmid #12259) were used. To generate pLJM1-EGFP transfer plasmids, four copies of inhibitory peptide or control peptide with mutated binding motif spaced out by a flexible GST linker and fused to C-terminus of EGFP were obtained (GenScript). At 72 h post transfection, the supernatants from cells transfected with lentivirus plasmids were filtered and stored at −80 °C. Potential adverse effects of the lentiviral constructs on cell growth were evaluated. Fifty thousand cells were seeded in 12-well plates and the number of cells was counted every 24 h using a countess II fl automated cell counter (Invitrogen).

### Viral infections

VeroE6 or VeroB4 cells were seeded into greiner CELLSTAR® 96-well plates containing EGFP-PABPi mut or EGFP-PABPi lentivirus (Fig. 6F) in DMEM containing 2% FBS and 1 μg/mL polybrene, and incubated for 72 h. Transduced cells were then infected with a panel of RNA viruses (VeroE6: SARS-2 (MOI: 0.05 for 16 h), JEV (MOI: 0.1 24 h), WNV (MOI: 0.1 24 h), YFV (MOI: 0.1 24 h), ZIKV (MOI: 0.1 24 h), RVFV (MOI: 0.05 for 16 h), VSV (MOI: 0.001 5 h), SINV (MOI: 0.05 for 16 h) and CHIKV (MOI: 0.05 for 16 h), VeroB4: DENV, TBEV and LGTV with MOI:0.1 for 24 h. Virus was detected using the following primary antibodies, SARS-2 (SARS-CoV-2 nucleocapsid (Rabbit monoclonal, Sino Biological Inc., 40143-R001)), JEV, WNV, DENV, and ZIKV (mouse monoclonal anti-flavivirus E HB112 ATCC), YFV (YFV E CRC 1689 ATCC), TBEV and LGTV (mouse monoclonal anti-TBEV E 1786, PMID: 7817895[RL1]), VSV, SINV, and CHIKV (mouse monoclonal to J2 (Scicons 10010500)), and secondary antibodies either donkey anti-mouse or donkey anti-rabbit IgG Alexa Fluor 555 secondary antibody (Invitrogen). Nuclei were counterstained by DAPI. Number of infected cells were determined using a TROPHOS Plate RUNNER HD® (Dioscure, Marseille, France). Number of infected cells were normalized to DAPI count and presented as percentage infection of mutated peptide.

### Viral titrations

SARS-CoV-2 was diluted in ten-fold dilutions and added to VeroE6 cells followed by 1 h incubation. The inoculum was replaced with an overlay containing DMEM, 2% FBS, 1% PEST, and 1.2% Avicel. After 24 h of infection cells were fixed in 4% formaldehyde for 30 min, permeabilized in PBS 0.5% trition-X-100 and 20 mM glycine. Viral foci were detected using primary monoclonal rabbit antibodies directed against SARS-CoV-2 nucleocapsid (Sino Biological Inc., 40143-R001), and secondary anti-rabbit HRP conjugated antibodies (1:2000, Thermo Fisher Scientific). Viral foci were then revealed by incubation with TrueBlue peroxidase substrate for 30 min (KPL, Gaithersburg, MD). TBEV was titrated as previously described^116^. VeroB4 cells were infected with 10-fold serial dilutions of TBEV. After 48 h of infection, cells were fixed with 4% formaldehyde and permeabilized in PBS containing 0.5% Triton X-100 and 20 mM glycine. Viral foci were detected using primary mouse antibodies directed against TBEV followed by staining with a horseradish peroxidase-conjugated anti-mouse secondary antibody (1:2000, Thermo Fisher Scientific).

### Immunofluorescence microscopy of EGFP-PABPi transfected cells

VeroB4 cells expressing either EGFP-PABPi mut or EGFP-PABP inhibitor peptides (EGFP-PABPi) were seeded in 8-well chamber slides (Sarstedt) and infected with TBEV at an MOI of 1 for 24 h. The cells were fixed with 4% formaldehyde and incubated with permeabilization buffer (0.3% Triton X-100 and 1% Goat serum in PBS) containing primary antibodies against dsRNA J2 ((1:1000) Scicons 10010500) and PABPC1 ((1:100) Abcam ab21060) followed by incubation with DAPI (1:1000) and conjugated secondary antibodies anti-mouse Alexa555 and anti-rabbit Alexa647 (1:500, Thermo Fisher Scientific). Coverslips were mounted and samples were analyzed using a Leica SP8 Laser Scanning Confocal Microscope with a 63x oil objective (Leica) and Leica Application Suit X software (LAS X, Leica). For the quantification of the RadialCV a total of 6 images containing 185 and 158 infected cells from EGFP-PABPi mut and EGFP-PABPi, respectively, were analyzed using CellProfiler. The DAPI channel was used to identify the nuclei as primary objects while the PABPC1 channel was used to identify the whole cells as secondary objects. These two objects where then used to identify the cytoplasmic fraction as a tertiary object. The cytoplasmic fraction was analyzed with the “MeasureObjectIntensity” and “MeasureObjectIntensityDistribution” functions to determine infected cells using the dsRNA integrated intensity and create the fractions within the cytoplasm to determine the distribution of dsRNA signal using the RadialCV.

### AP-MS

The growth media contained 10% FBS (Gibco), non-essential amino acids (NEAA, Gibco), and 5 µg/mL and 5 units/mL penicillin-streptomycin (Gibco). One T175 flask of HEK293 cells of 70% confluency per condition was transiently transfected using 90 µg of EGFP-PABPi or EGFP-PABPi mut, and Lipofectamine 3000 (Invitrogen) according to manufacturer’s instructions. The cells were harvested 24 h after transfection by first washing with ice-cold DPBS (Gibco) then scraped into 3 ml ice-cold lysis buffer (10 mM Tris-HCl, pH 7.5, 150 mM NaCl, 1% NP-40 substitute (Sigma 74385), 1x Protease inhibitor (Roche, cOmplete, Mini, EDTA-free, 4693159001) and incubated on ice for 30 min while shaking. The lysate was clarified by centrifugation at 16000 g for 15 min at 4 °C. Similarly prepared, but SARS-CoV-2 or TBEV infected VeroE6 or VeroB4 cells, respectively, were also used, stably expressing the above-mentioned constructs. The protein concentration was determined using DC Protein Assay (Bio-Rad).

The cell lysate was diluted to 0.8 mg protein/ml with dilution buffer (10 mM Tris-HCl, pH 7.5, 150 mM NaCl, 1x Protease inhibitors), and 1 mg protein was used per replicate. Cell lysates were incubated with GFP-Trap® Dynabeads™ (Chromotek) at 4 °C for 1 h while rotated. After washing, the interacting proteins were eluted using acidic elution buffer (200 mM glycine-HCl, pH 2.5) and neutralized with 1 M ammonium bicarbonate instantly. The eluate was reduced with DTT and alkylated with IAA, then digested overnight using trypsin at 37 °C. The digestion was stopped using an acidifying solution (83.3% AcN, 16.7% TFA) to pH < 3. The peptides were desalted using STageTips made in-house^117,118^, with centrifugal elution. Briefly, 2 layers of C18 membrane (3 M Empore) were placed in a 200 µl pipette tip, activated with methanol and 80% AcN, 0.1% formic acid, then washed twice with 0.1% formic acid. After that the acidified samples were loaded, washed with 0.1% formic acid, and eluted with 80% AcN, 0.1% formic acid. The eluted sample was vacuum-dried and stored at −80 °C.

The samples were analyzed using an Easy-nLC 1000 nanoLC (Thermo) with an Acclaim PepMap 100 pre-column (Thermo, 75 µm × 2 cm, 3 µm, 100 Å) and a PepMap RSLC C18 analytical column (Thermo, EASYspray, 75 µm × 15 cm, 2 µm, 100 Å). The mass spectrometer was a QExactive Plus Orbitrap instrument (Thermo) equipped with an EASYspray ion source. For peptide separation, a gradient method was applied, where the gradient went from 4 to 76% acetonitrile in 79 min. The MS was operated in the positive ion mode with a resolution of 140,000 for full scan (400–1700 *m*/*z*), and 17500 for MS/MS with the automatic gain control (AGC) target of 3 × 10^6^ and 1 × 10^5^, respectively. The ESI spray voltage was 1.9 kV. Data-dependent acquisition was used, with the top 10 most abundant ions fragmented and measured in MS/MS. Dynamic exclusion of 30 s was enabled.

The raw files were analyzed using MaxQuant (version 2.0.1.0) using FASTA files acquired from Uniprot: *Homo sapiens* (2022.02.21, reviewed, 20360 entries) for HEK293 samples and *Chlorocebus* (2022.02.22, reviewed and unreviewed, 20717 entries) for VeroE6 and VeroE4 samples with or without proteins of the SARS-CoV-2 variant patient isolate SARS-CoV-2/01/human/2020/SWE accession no/GeneBank no MT093571.1 or TBEV Torö−2003, GenBank Accession no. DQ401140.3. Trypsin/P was selected as the digestion enzyme, with maximum 2 missed cleavages allowed. For variable modifications methionine oxidation and N-terminal acetylation were allowed, while for fixed modification carbamidomethylation of cysteines was selected. Label-free quantification was chosen using the MaxLFQ algorithm^119^ and a minimum ratio count of two. The used peptide mass tolerances were 20 and 4.5 ppm for first and main search, respectively. PSM and protein FDR was set to 0.01. The minimum number of detected peptides was set to 2, and the minimum number of unique peptides to 1 for identification.

To identify interacting proteins, the data was processed first with Perseus (2.0.3.0)^120^. Using the proteingroups.txt result file from MaxQuant, the possible contaminants, reverse hits and proteins only identified by site were removed. The LFQ intensities were transformed to a log_2_(x) base, and the hits were filtered, only keeping rows with at least 3 valid values in at least one of the categorical groups (sample/control). The missing values were replaced from normal distribution with a width of 0.3 and down shift of 1.8 (mode: total matrix). Two-sided t-test was used for significance testing (*p*-value <0.05, S0:0) and the results were visualized in a Volcano plot using a fold-change cut off of 2. Results are also found in Supplementary data 10.

### Reporting summary

Further information on research design is available in the Nature Portfolio Reporting Summary linked to this article.

## Supplementary information

Supplementary Information

Description of Additional Supplementary Files

Reporting Summary

Supplementary Data 1

Supplementary Data 2

Supplementary Data 3

Supplementary Data 4

Supplementary Data 5

Supplementary Data 6

Supplementary Data 7

Supplementary Data 8

Supplementary Data 9

Supplementary Data 10

## Data Availability

The interaction data generated through proteomic peptide-phage display in this study are provided in Supplementary data 3. The protein interactions from this publication have also been submitted to the IMEx (http://www.imexconsortium.org) consortium through IntAct^25^ under the identifier IM-29580. The mass spectrometry proteomics data generated in this study have been deposited in the ProteomeXchange Consortium via the PRIDE (Perez-Riverol et al. (2022)) partner repository under accession code PXD033874. The crystal structures have been deposited in PDB and are available with the PDB is 7BN1, 7BN2, and 7BN3. Source data are provided with this paper.

## Source data

Source data

## Code availability

Relevant code has been made available (https://zenodo.org/deposit/6583610).
