# Supplementary material for: Large-scale phage-based screening reveals extensive pan-viral mimicry of host short linear motifs
Source: Nat Commun. 2023 Apr 26;14:2409. doi: 10.1038/s41467-023-38015-5 (PMC10132805; doi:10.1038/s41467-023-38015-5)
Supplement: Supplementary file 1 — Supplementary Information [file 41467_2023_38015_MOESM1_ESM.pdf]

Large-scale phage-based screening reveals extensive pan-viral mimicry of host short linear motifs

Mihalic et al., 2023

SUPPLEMENTAL FIGURES AND TABLES

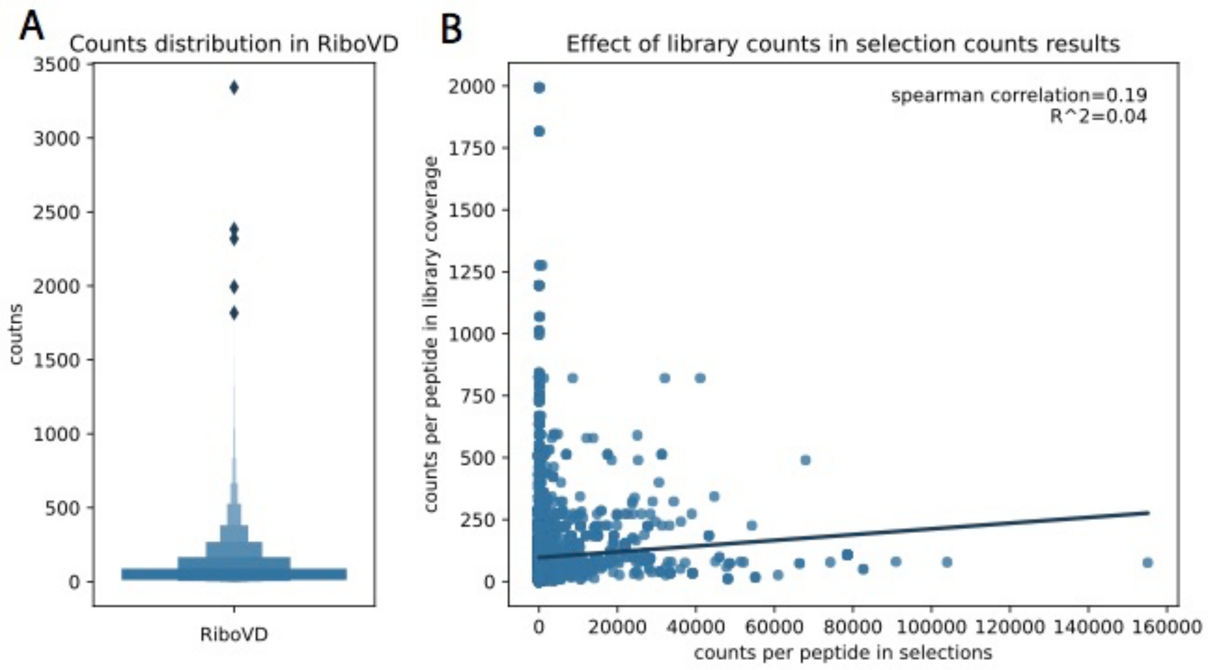

Figure S1. **RiboVD counts distribution and effect on selections.**

(**A**) RiboVD peptides count distribution (sequencing of naive library). (**B**) Correlation analysis between RiboVD peptides counts in coverage vs. medium-/high-confidence peptides counts in selections.

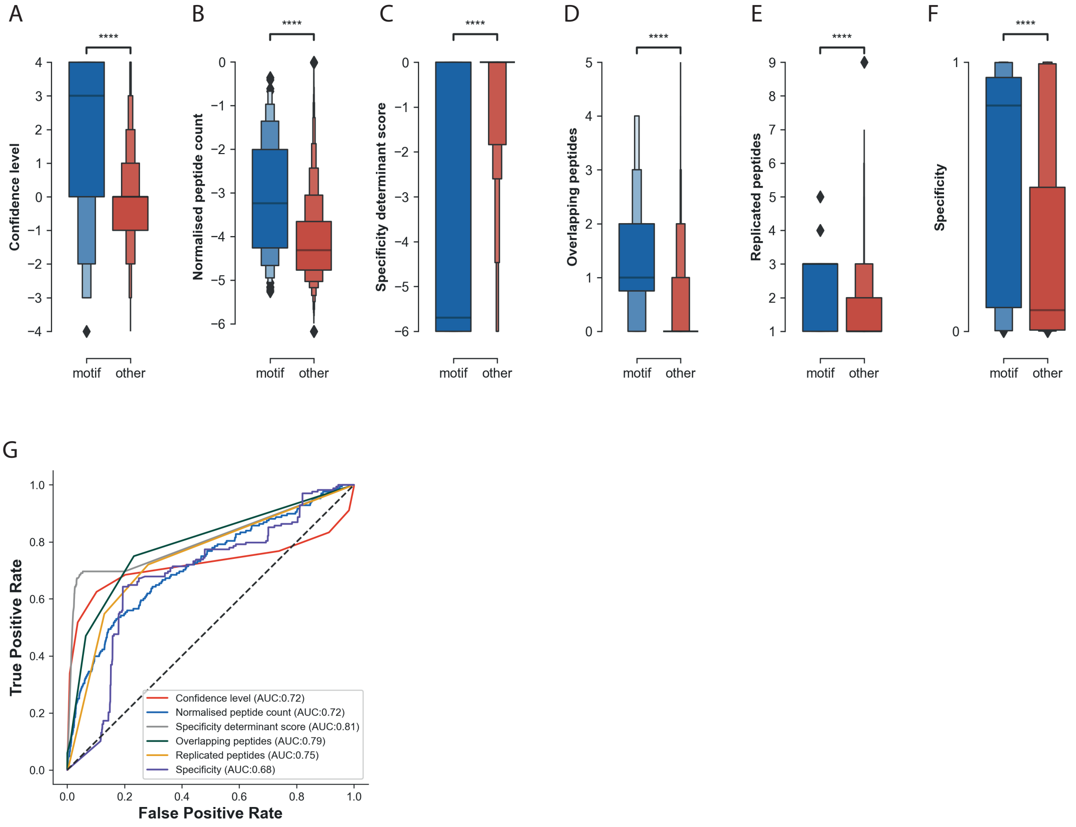

Figure S2. Evaluation of metrics for assignment of confidence levels.

(**A-F**). Boxen plot of motif-containing peptides from the benchmarking datasets (blue) compared (Mann–Whitney U test) to all other selected peptides (red) for (**A**) consensus confidence level (defined based on the replicated peptides, overlapping peptides, specificity determinant match, and normalized peptide count), (**B**) log10 of the normalized peptide count, (**C**) log10 of PSSM-derived specificity determinant score defining the similarity of the selected peptides to the SLiMFinder-discovered enriched motif (only available for baits with an enriched motif), (**D**) overlapping peptides, (**E**) replicated peptides, and (**F**) the specificity score. n_{motif} =168 and n_{other} =84,919 for all boxenplots with the exception of specificity in panel F where n_{motif} =117 and n_{other} =17,039. Asterisks denote the likelihood of the null hypothesis that the distribution underlying each sample is the same using a Mann–Whitney U test (****P-value = < 1.0 × 10−4). Boxen plots (B-F) are used to more accurately visualize the distribution of values. The central section has two blocks each containing 25% of the data split by the median (denoted by a dark black bar) and each additional block represents 50% of the data of the previous block. (**G**) ROC curves of the metrics to assign confidence levels.

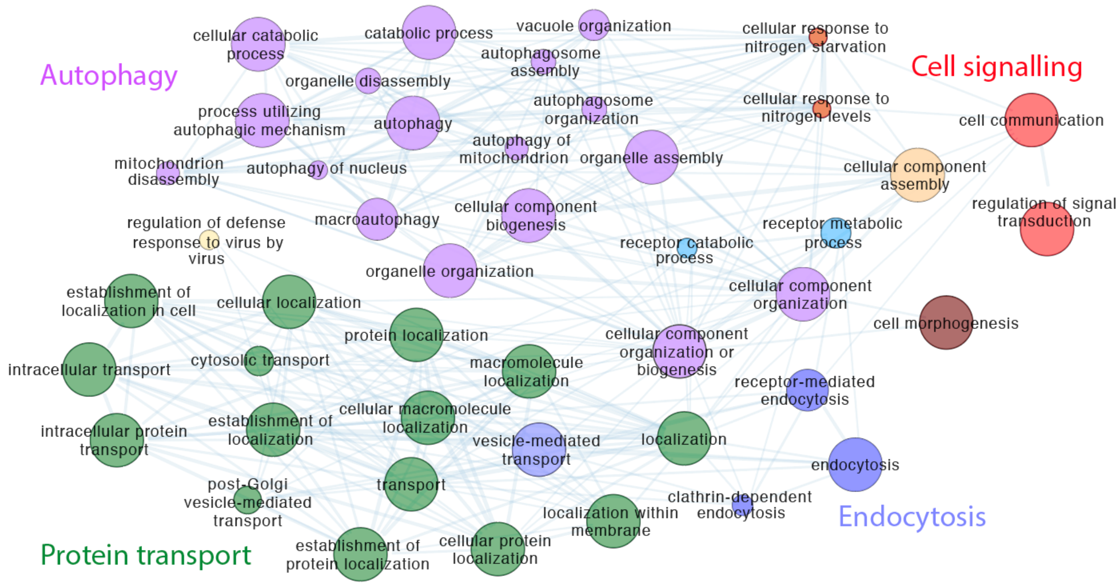

Figure S3. **GO Biological Process term enrichment of the expanded host-virus interaction network**.

Edges represent overlapping genes between the terms, Node size increases with the percentage of genes annotated with the specific term are found in the network, colors correspond to the respective annotated processes on the figure.

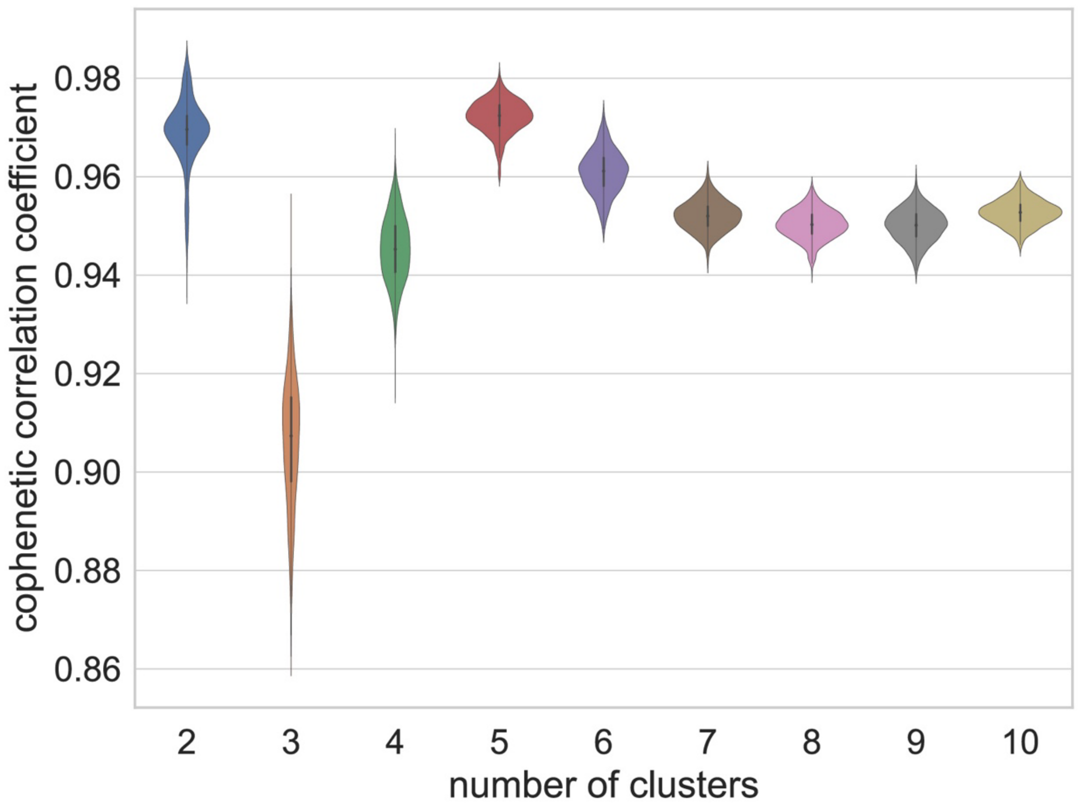

Figure S4. Cophenetic correlation coefficient for different numbers of clusters (latent factors) to determine the numbers of clusters that provides robust cluster membership. The distribution of the cophonetic correlation coefficient was derived from n=1000 iterations of the NMF as described in the Methods.

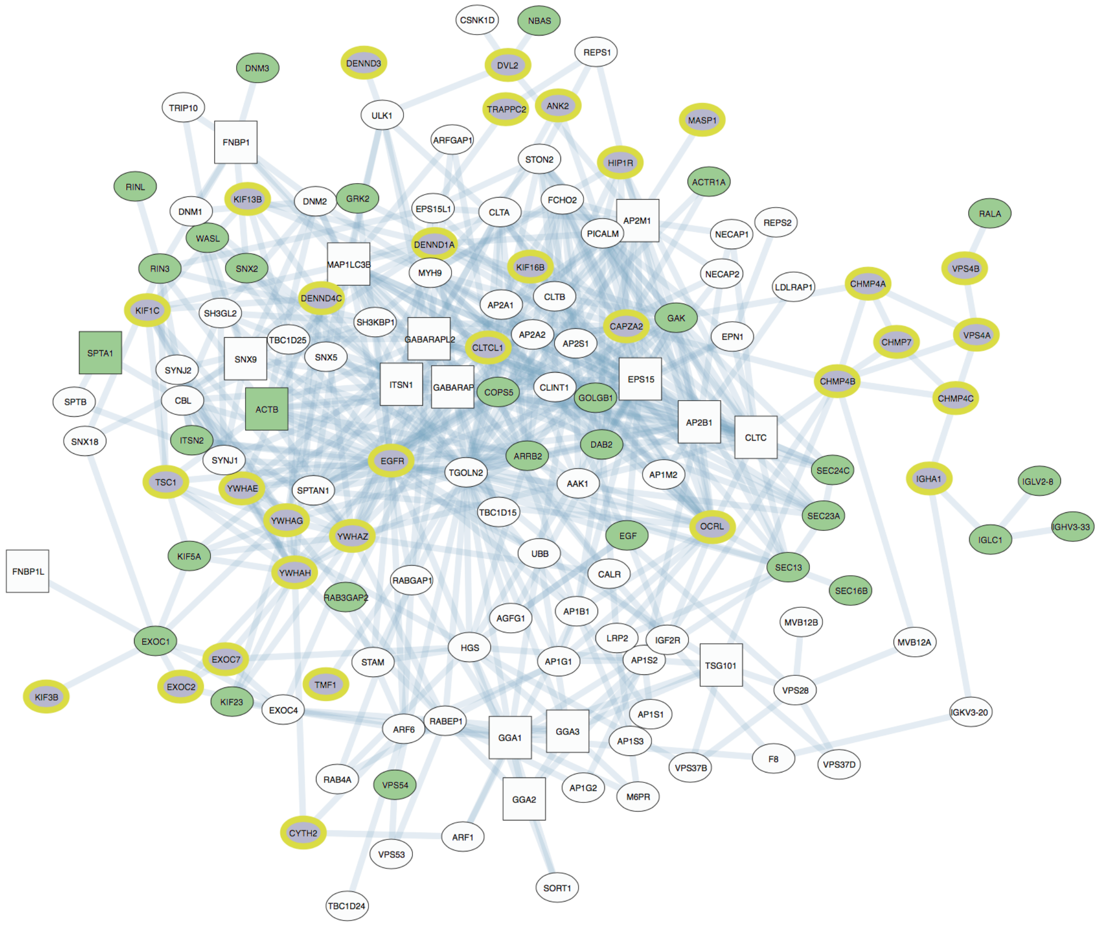

Figure S5. **Differences in the vesicle-mediated transport networks targeted by viruses in cluster 4 (green) vs those in cluster 5 (lilac/yellow).** White nodes are common to both target networks. Squares indicate proteins used as baits in the experiment.

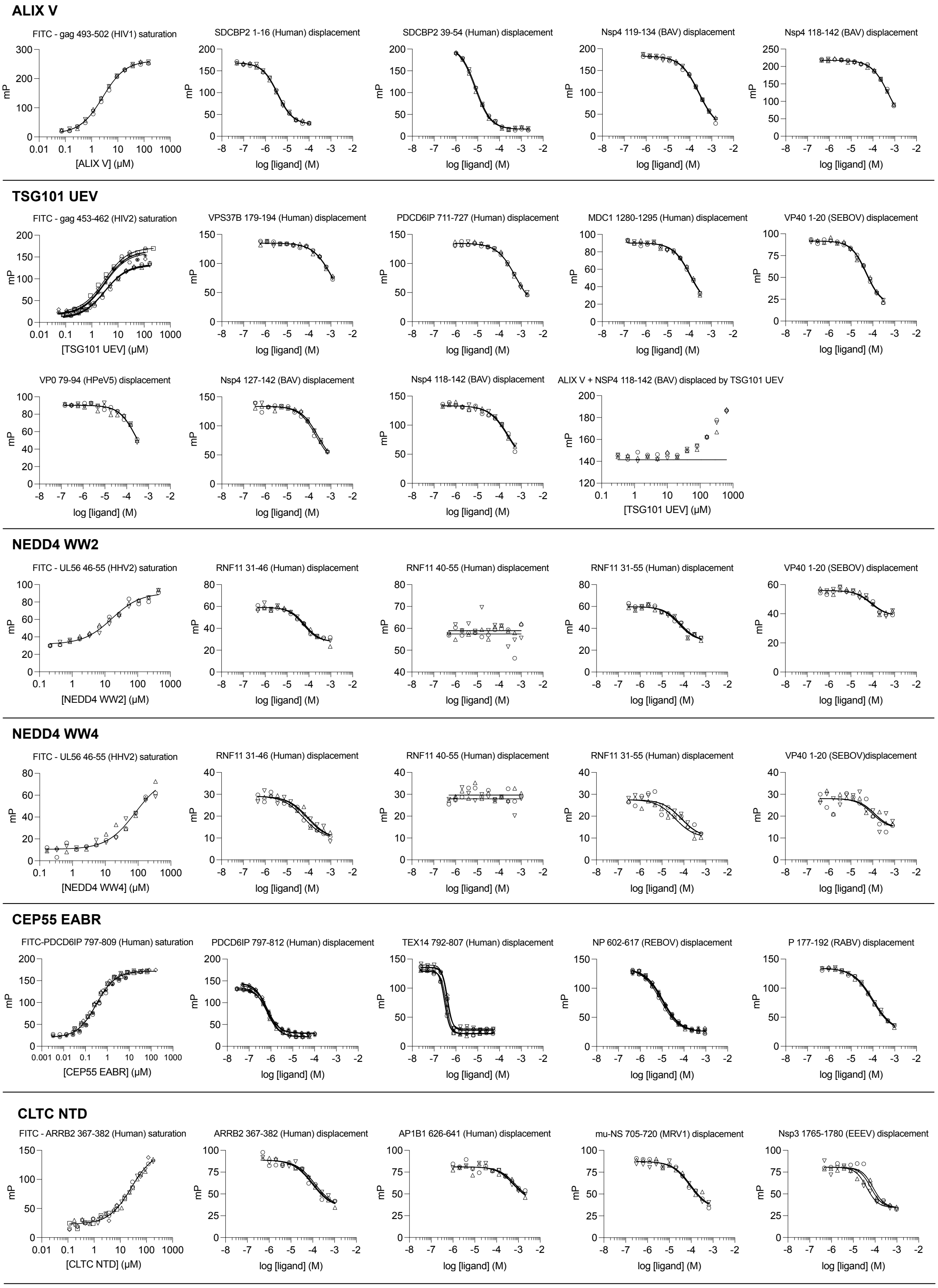

ALIX V
FITC - gag 493-502 (HIV1) saturation
SDCBP2 1-16 (Human) displacement
SDCBP2 39-54 (Human) displacement
Nsp4 119-134 (BAV) displacement
Nsp4 118-142 (BAV) displacement
mP
[ALIX V] (μM)
log [ligand] (M)
TSG101 UEV
FITC - gag 453-462 (HIV2) saturation
VPS37B 179-194 (Human) displacement
PDCD6IP 711-727 (Human) displacement
MDC1 1280-1295 (Human) displacement
VP40 1-20 (SEBOV) displacement
[TSG101 UEV] (μM)
VP0 79-94 (HPeV5) displacement
Nsp4 127-142 (BAV) displacement
Nsp4 118-142 (BAV) displacement
ALIX V + NSP4 118-142 (BAV) displaced by TSG101 UEV
NEDD4 WW2
FITC - UL56 46-55 (HHV2) saturation
RNF11 31-46 (Human) displacement
RNF11 40-55 (Human) displacement
RNF11 31-55 (Human) displacement
VP40 1-20 (SEBOV) displacement
[NEDD4 WW2] (μM)
NEDD4 WW4
FITC - UL56 46-55 (HHV2) saturation
RNF11 31-46 (Human) displacement
RNF11 40-55 (Human) displacement
RNF11 31-55 (Human) displacement
VP40 1-20 (SEBOV)displacement
[NEDD4 WW4] (μM)
CEP55 EABR
FITC-PDCD6IP 797-809 (Human) saturation
PDCD6IP 797-812 (Human) displacement
TEX14 792-807 (Human) displacement
NP 602-617 (REBOV) displacement
P 177-192 (RABV) displacement
[CEP55 EABR] (μM)
CLTC NTD
FITC - ARRB2 367-382 (Human) saturation
ARRB2 367-382 (Human) displacement
AP1B1 626-641 (Human) displacement
mu-NS 705-720 (MRV1) displacement
Nsp3 1765-1780 (EEEV) displacement
[CLTC NTD] (μM)

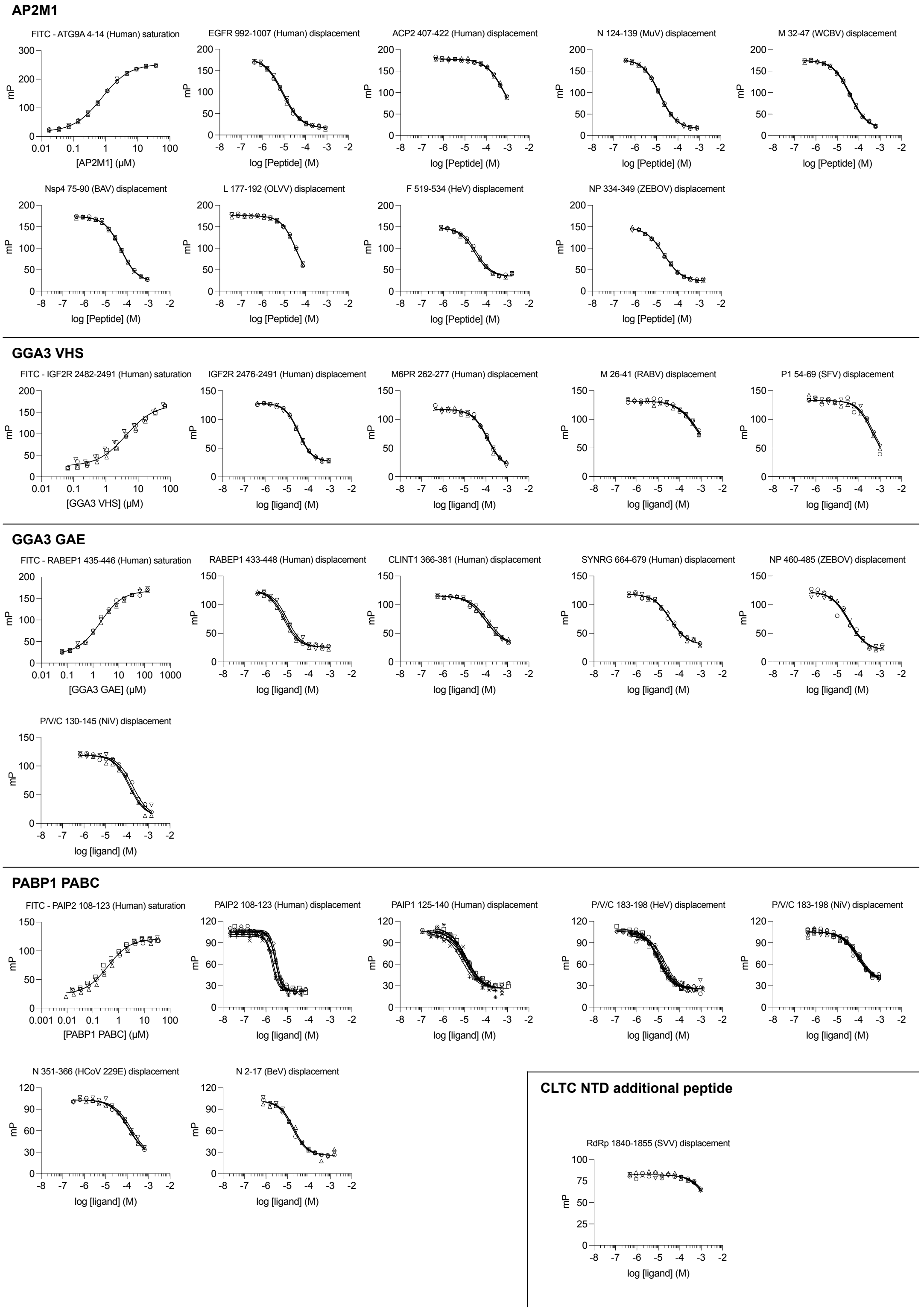

Figure S6. **All FP affinity measurements performed in this study.** Peptides used, and calculated affinities are presented in Table S8. Source data are provided as a Source Data file.

MDSRPQKIWMAPSLTESDMDYHKILTAGLSVQQGIVRQRVIPVYQVNNLEEICQLIIQAFEAGVDFQESA
DSFLLMLCLHHAYQGDYKLFLESGAVKYLEGHGFRFEVKKRDGVKRLEELLPAVSSGKNIKRTLAAMPEE
ETTEANAGQFLSFASLFLPKLVVGEKACLEKVQRQIQVHAEQGLIQYPTAWQSVGHMMVIFRLMRTNFLI
KFLLIHQGMHMVAGHDANDAVISNSVAQARFSGLLIVKTVLDHILQKTERGVRLHPLARTAKVKNEVNSF
KAALSSLAKHGEYAPFARLLNLSGVNNLEHGLFPQLSAIALGVATAHGSTLAGVNVGEQYQQLREAATEA
EKQLQQYAESRELDHLGLDDQEKKILMNFHQKKNEISFQQTNAMVTLRKERLAKLTEAITAASLPKTSGH
YDDDDDIPFPGPINDDDNPGHQDDDPTDSQDTTIPDVVVDPDDGSYGEYQSYSENGMNAPDDLVLFDLDE
DDEDTKPVPNRSTKGGQQKNSQKGQHIEGRQTQSRPIQNVPGPHRTIHHASAPLTDNDRRNEPSGSTSPR
MLTPINEEADPLDDADDETSSLPPLESDDEEQDRDGTSNRTPTVAPPAPVYRDHSEKKELPQDEQQDQDH
TQEARNQDSDNTQSEHSFEEMYRHILRSQGPFDAVLYYHMMKDEPVVFSTSDGKEYTYPDSLEEEYPPWL
TEKEAMNEENRFVTLDGQQFYWPVMNHKNKFMAILQHHQ

Figure S7. **Zaire ebolavirus Nucleoprotein sequence (Uniprot entry P18272**).
All of the instances matching the AP2M1 recognition motif as reported in eukaryotic linear motif database (ELM) are highlighted in blue. The AP2M1 interaction motif identified in this study is highlighted in green and the GGA3 GAE motif is highlighted in magenta.

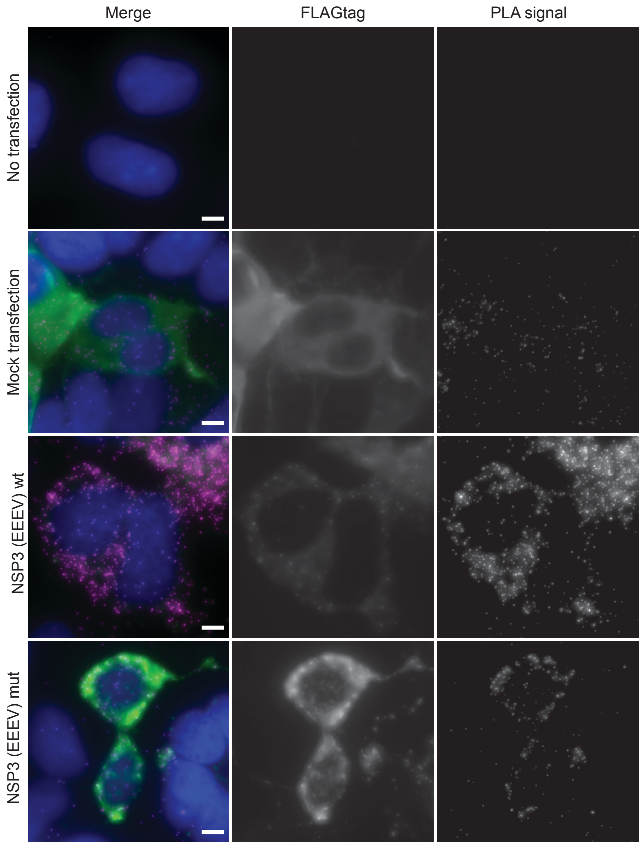

Figure S8. Proximity ligation assay probing the interaction between endogenous clathrin and full-length FLAG-tagged NSP3 (EEEV) in HEK293 cells. Fluorescence microscopy images. Nuclei are in blue, FLAG-tag in green and PLA signals visualizing clathrin-NSP3 (EEEV) interaction in magenta. Scale bar is 5 μm.

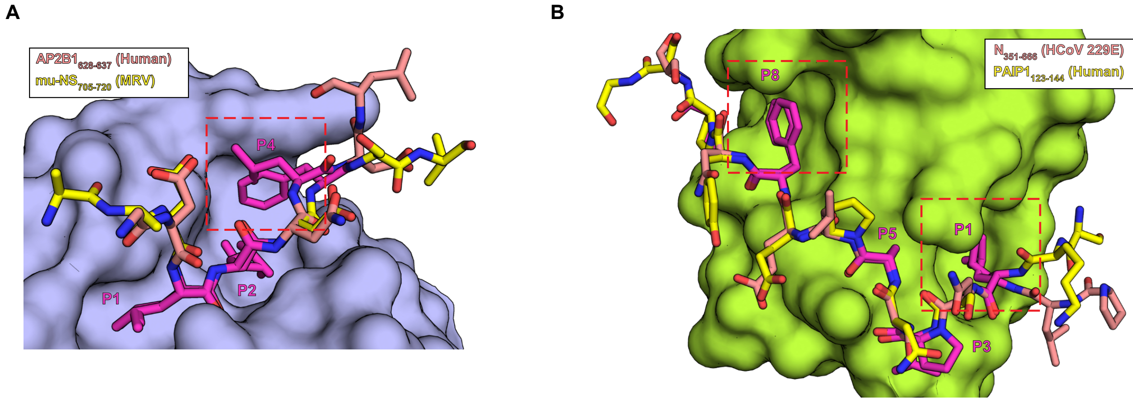

Figure S9. Viral and human ligands bind the CLTC NTD and PABP1 PABC in similar conformation. (A) Crystal structure model of CLTC NTD with superimposed viral ligand from mu-NS (MRV) in yellow and the human ligand from AP2B1 (PDBid: 5M5R) in salmon. The red rectangle highlights the different position of Phe and Leu in position P4. **(B)** Crystal structure model of PABP1 PABC with superimposed viral ligand from N (HCoV 229E) in salmon and the human ligand from PAIP1 (PDBid: 3NTW) in yellow. The red rectangles highlight the two hydrophobic binding pockets at position P1 and P8

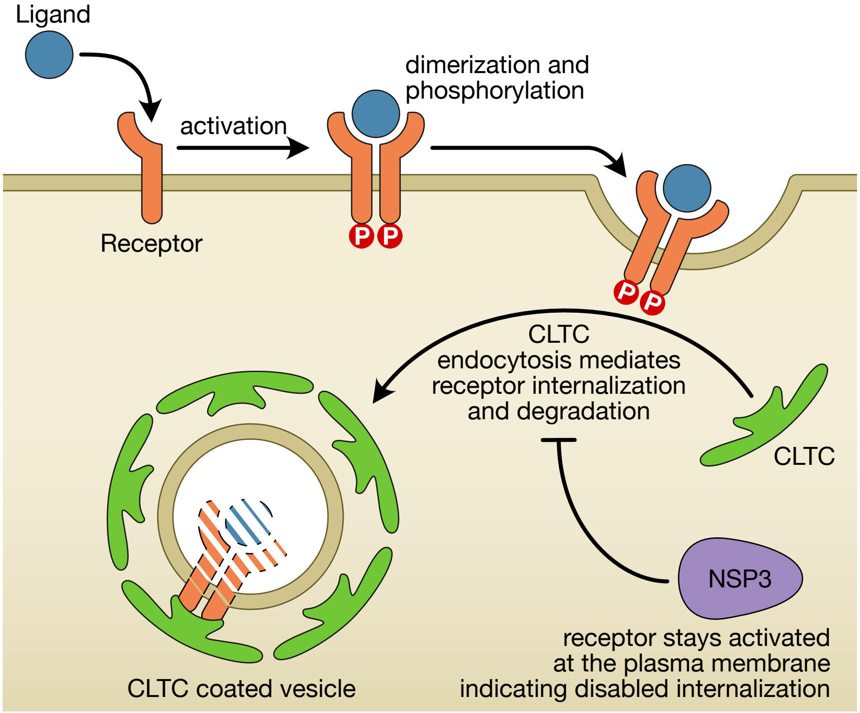

Figure S10. Generalized model of the NSP3 (EEEV) effect on receptor internalization. Upon ligand binding the receptor dimerizes, is activated via autophosphorylation and subsequently internalized from the plasma membrane via clathrin mediated endocytosis. The presence of NSP3 (EEEV) causes the receptor to remain at the plasma membrane in its activated form indicating that clathrin-mediated endocytosis is not functioning properly.

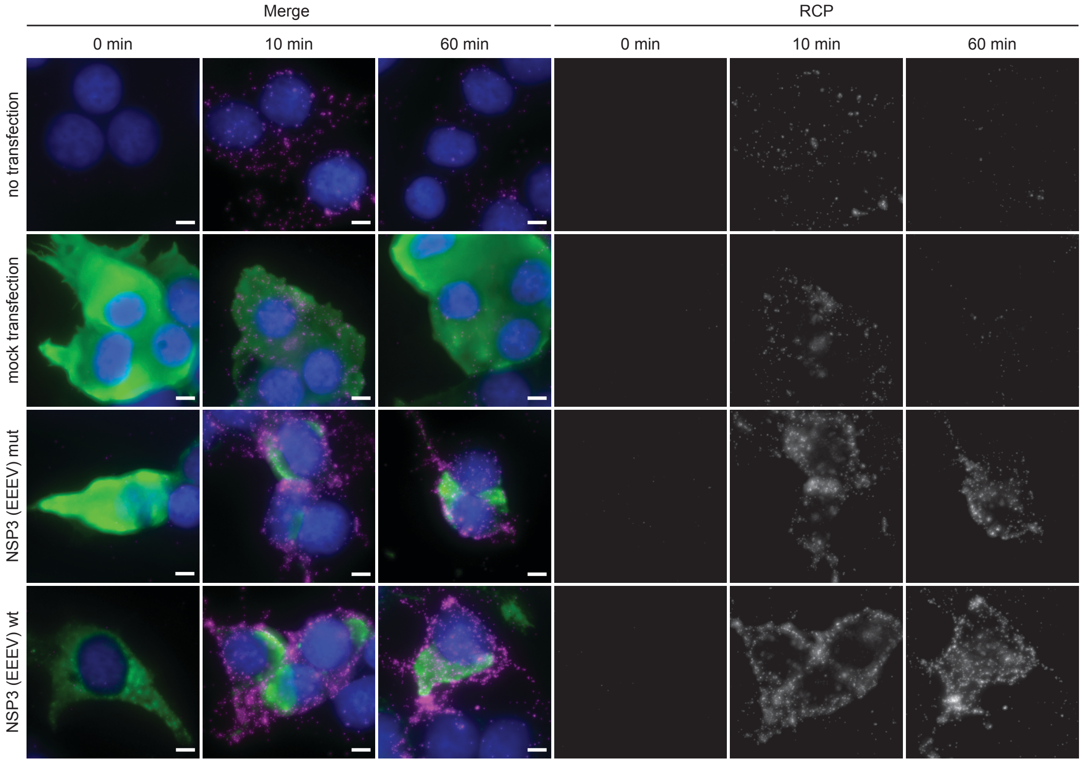

Figure S11. Proximity ligation assay probing the activation of PDGFRβ in HEK293-PDGFRβ-HA cells. Fluorescence microscopy images. Nuclei are in blue, FLAG-tag in green, and PLA signals visualizing phosphorylated PDGFRβ in magenta. Scale bar is 5 μm. Six biological replicates were performed.

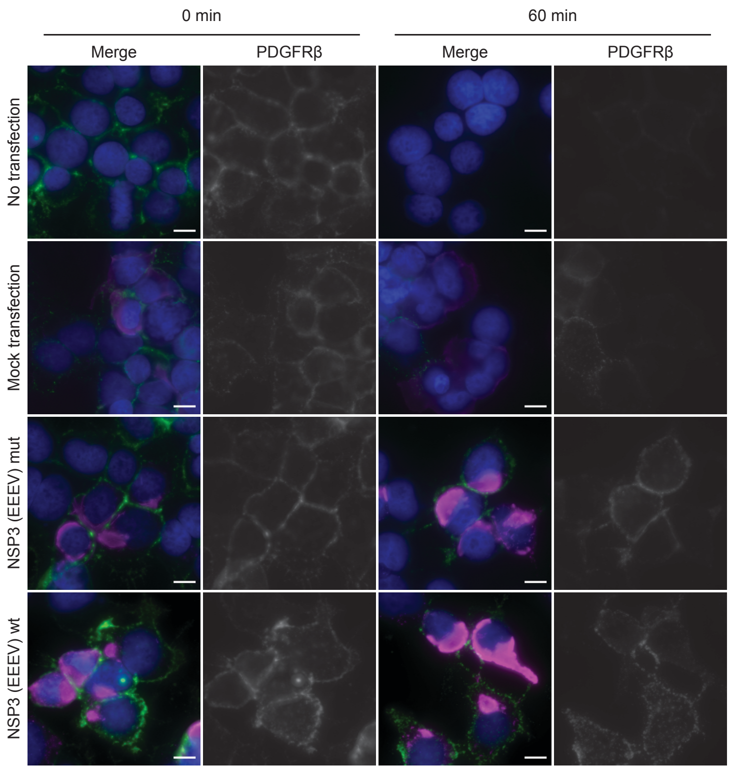

Figure S12. Cell surface fluorescence probing the extracellular part of PDGFRβ. Fluorescence microscopy images. Nuclei are in blue, FLAG-tag is in magenta, and PDGFRβ is in green. Scale bar is 5 µm. Integrated fluorescence intensity was measured over 3 biological replicates

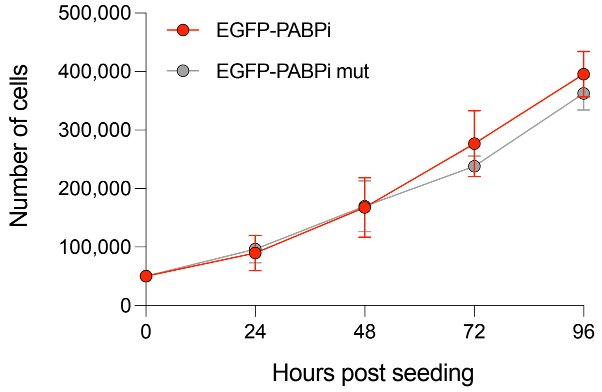

Figure S13. Cell growth curve of VeroE6 cells expressing EGFP-PABPi or EGFP-PABPi mut. Fifty thousand cells were seeded in 12-well plates and the cells were counted every 24 hours for four days. The data are presented as means ± SD and were done in 6 biological replicates

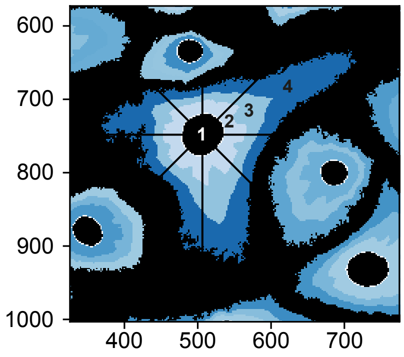

Figure S14. Schematic representation of the measurement of signal distribution and localization in the cell shown (related to Figure 6J). The distance and radius of the dsRNA replication complexes are detected and grouped in 4 fractions.

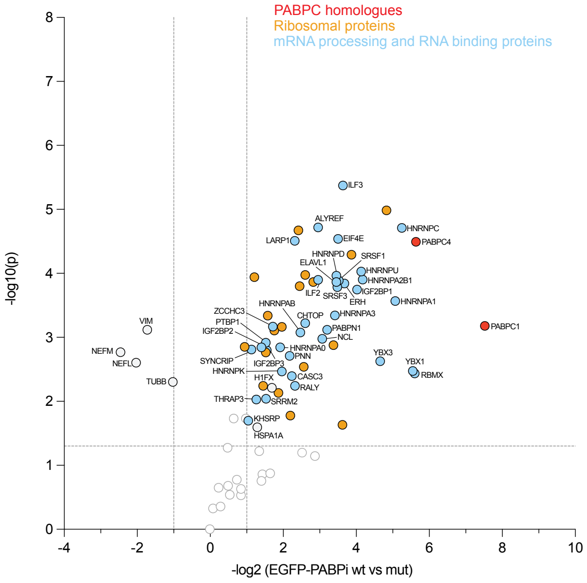

Figure S15. AP-MS results of EGFP-PABPi pulldown from HEK293 cells. Source data for is accessible in PRIDE database under accession PXD033874.

SUPPLEMENTAL TABLES

Table S1. Overview of crystallographic data.

	**CLTC-EEV**	**CLTC-mu-NS**	**PAPBC1-HCoV**
**Data Collection**			
Space group	C2	C2	$C222_1$
a, *b*, *c* (Å)	136.9, 129.1, 77.9	137.53, 128.9, 78.10	63.51, 150.1, 65.5
α, β, γ (°)	90, 115.3, 90	90.00 115.5 90.00	90.0 90.0 90.0
Molecules in a. u.	2	2	3
Wavelength (Å)[a]	0.9795	0.9795	1.003
Resolution (Å)	70.41-1.96 (1.96-2.11)	70.49-1.97 (1.97-2.11)	43.63-1.93 (1.93-2.0)
Total reflections	214797 (10220)	216228 (10833)	312878 (31260)
Unique reflections	62270 (3115)	61571 (3080)	23731 (2268)
Multiplicity	3.4 (3.3)	3.5 (3.5)	13.2 (13.8)
Completeness (%)	90.2 (36.2)	93.3 (65.2)	99.1 (98.5)
$< I/\sigma(I) >$	10.0 (1.6)	9.1 (1.4)	12.7 (1.3)
Wilson B factor (Å^2)	23.3	43.4	53.7
R_{merge} [b]	0.070 (0.673)	0.078 (0.815)	0.098 (0.052)
R_{meas}	0.083 (0.804)	0.093 (0.964)	0.106 (0.056)
R_{pim}	0.044 (0.434)	0.049 (0.512)	0.030 (0.016)
$CC_{1/2}$	0.998 (0.629)	0.991 (0.561)	0.999 (0.999)
**Refinement**			
No. of reflection in work set	59020	58624	23713
No. of reflection in free set	3160	2950	1124
R_{work} [c]	0.1800	0.1854	0.1980
R_{free} [d]	0.2170	0.2189	0.2378
No. of non-hydrogen atoms			
total	6354	6293	2268
Protein	5687	5691	1858
Solvent	506	470	119
Peptide	161	132	269
Other ligands	-	-	19 (SO_4)
RMS deviations			
bonds (Å)	0.009	0.013	0.007
angles (°)	1.57	1.3	0.788
Residues in Ramachandran plot regions (%)			
favored	98.49	99.0	99.3
allowed	1.51	1.0	0.4
outliers	0.00	0.00	0.4
Average B factor (Å^2)	44.33	55.68	53.12
Protein	35.81	41.93	48.5
Solvent	40.13	46.35	56.42
Peptide	42.58	49.8	54.46
PDB-ID	7BN2	7BN1	7BN3

[a] Values in parentheses are for the highest-resolution shell

[b] $R_{merge} = \sum |I - \langle I \rangle| / \sum (I)$ I where is the observed integrated intensity, $\langle I \rangle$ is the average integrated intensity obtained from multiple measurements, and the summation is over all observed reflections.

[c] $$R\text{-}factor = \frac{\sum_{(h,k,l)} \| F_{obs}(h,k,l)| - |F_{calc}(h,k,l)\|}{\sum_{(h,k,l)} |F_{obs}(h,k,l)|}$$ where F_{obs} and F_{calc} are observed and calculated structure factors respectively.

[d] R_{free} was calculated as for R_{work} but only 5% data left out of refinement procedure has been used in the calculation

Table S2. Reagent and resource table.

Reagent/resource	Reference or source	Identifier, catalog number or reference
**Mammalian cells, bacteria, viral strains**		
HEK293	Sigma	85120602
HEK293T	TakaraBio	Z2180N (632180)
HEK293-PDGFRβ-HA	Kind gift from Frank Böhmer	[1,2]
VeroB4	Kind gift of Gerhard Dobler Bundeswehr Institute of Microbiology, Munich, Germany	
VeroE6	Kind gift of Mattias Forsell Umeå University	
SARS CoV-2	Public Health Agency of Sweden	SARS-CoV-2/01/human2020/SWE accession no/GeneBank no MT093571.1
JEV	Public Health Agency of Sweden	Nakayama strain
WNV	Public Health Agency of Sweden	WNV_0304h_ISR00
YFV	Public Health Agency of Sweden	Asibi
DENV	Public Health Agency of Sweden	serotype-2; PNG/New Guinea C
TBEV		Torö-2003[3]
LGTV	Kind gift of Gerhard Dobler Bundeswehr Institute of Microbiology, Munich, Germany	TP21
ZIKV	Kind gift of Gerhard Dobler Bundeswehr Institute of Microbiology, Munich, Germany	MR766
RVFV		Katushka[4]

VSV	Kind gift of Friedemann Weber, University of Freiburg	
SINV	Kind gift of Olivia Wesula Luande and Magnus Evander	Lovanger, KF737350
CHIKV	Kind gift of Magnus Evander	CHIKV LR2006OPY1
E.coli OmniMAX	Thermo Fisher Scientific	C854003
E.coli gold BL21 (DE3)	Agilent technology	230132
NEB® Stable Competent *E. coli*	New England Biolabs	C3040I
**Recombinant DNA**		
pLJM1-EGFP	David Sabatini lab	Addgene plasmid #19319
psPAX2	Didier Trono lab	Addgene plasmid #12260
pMD2.G	Didier Trono lab	Addgene plasmid #12259
**Antibodies**		
Goat-anti-FLAGtag	abcam	ab1257
Mouse-anti-FLAG M2	Sigma Aldrich	F1804
mouse-anti-clathrin	abcam	ab2731
Goat-anti-GST	Cytiva	274577012
rabbit-anti-PDGFRβ	Cell Signaling Technology	#3169
mouse-anti-PDGFRβ-pY751	Cell Signaling Technology	#3166
Donkey anti-Goat IgG (H+L) Highly Cross-Adsorbed Secondary Antibody, Alexa Fluor Plus 647	Invitrogen	A32849
Donkey anti-Rabbit IgG (H+L) Highly Cross-Adsorbed Secondary Antibody, Alexa Fluor Plus 555	Invitrogen	A32794
goat-anti PDGFRβ	RnD Systems	AF385
rabbit-anti-FLAGtag	Cell Signaling Technology	#14793S
rabbit anti-SARS-CoV-2 Nucleocapsid	Sino Biological Inc	40143-R001
mouse anti-TBEV E 19/1786		[5]

mouse anti-Flavivirus Group Antigen Antibody, clone D1-4G2-4-15	ATCC	HB-112
mouse anti-YFV E	ATCC	CRL 1689
mouse anti dsRNA J2	Scicons	10010500
rabbit anti-VSV-G	Sigma	V4888-200UG
donkey anti-Rabbit IgG (H+L) Highly Cross-Adsorbed Secondary Antibody, Alexa Fluor 555	Invitrogen	A-31572
donkey anti-Mouse IgG (H+L) Highly Cross-Adsorbed Secondary Antibody, Alexa Fluor 555	Invitrogen	A31570
Goat anti-Rabbit IgG (H+L) Secondary Antibody, HRP	Invitrogen	31460
Goat anti-Mouse IgG (H+L) Secondary Antibody, HRP	Invitrogen	31430
IRDye® 680RD Donkey anti-Goat IgG secondary Antibody	LI-COR	925-68074
IRDye® 800CW Goat anti-Mouse IgG Secondary Antibody	LI-COR	925-32210
**Oligonucleotides and sequence-based reagents**		
**Chemicals, enzymes and other reagents**		
Pierce™ Glutathione Agarose	Thermo Scientific	16100
Ni sepharose™ excel	Cytiva	17371201
KH_2PO_4	Alfa Aesar	7778-77-0
K_2HPO_4	VWR	7758-11-4
Peptone	Sigma Aldrich	91249
Yeast extract	Merck Millipore	1.03753.0500
DTT	Fisher bioreagents	3483-12-3
NaCl	VWR	27810.295
TCEP	Thermo Scientific	20491
Isopropyl-beta-D-thiogalactopyranoside	Biosynth Carbosynth	367-93-1
tris(hydroxymethyl)aminomethane	Merck Millipore	1.08382.1000

Imidazole	Merck Millipore	1.04716.1000
Kanamycin Sulfate	VWR	25389-94-0
Ampicillin	MEDA/Apoteket	011406
L-glutathion, reduced	Alfa Aesar	A18014.06
Mini-Protean TGX Stain-free gels	Bio Rad	4568096
RNase	Roche	10109134001
DNase I	Roche	10104159001
0.2 µm sterile filter	Sarstedt	83.1826.001
PreScission protease	Produced in-house	
Thrombin	Cytiva	27084601
M13KO5 helper phage	ThermoFisher	18311019
50 bp marker	Thermo Scientific	10416014
GelRed	Biotium	41003-T
QIAquick PCR Purification Kit	Qiagen	28104
Mag-Bind® TotalPure NGS	Omega Bio-Tek	M1378-00
DMEM with GlutaMAX Supplement	Gibco	61965026
DMEM/F-12	Gibco	11320033
FBS	Gibco	26140087
MEM Non-Essential Amino Acids Solution	Gibco	11140035
Penicillin-Streptomycin	Gibco	15070063
Lipofectamine 3000	Invitrogen	L3000008
Turbofect Transfection Reagent	Thermo Scientific	R0531
Polyethyleneimine, linear	Thermo Scientific	43896
DPBS	Gibco	14190094
NP-40 Substitute	Sigma	74385
cOmplete, EDTA free protease inhibitor	Roche	05056489001
DC assay kit	Bio-Rad	5000114/5000113/5000115
GFP-Trap Dynabeads	Chromotek	gtd-20
Lysozyme	ITW reagents	A4972
Phusion High-Fidelity polymerase	Thermo Fisher Scientific	F-530S
Trypsin	Promega	V5111sn

C18 membrane	3M Empore	2215
Opti-MEM	Gibco	11058021
Intercept (TBS) Blocking Buffer	Li-cor	927-60001
TrueBlue peroxidase substrate	KPL	KPLI50-78-02
Duolink PLA probe anti-Mouse PLUS	Olink	82021
Duolink PLA probe anti-Goat MINUS	Olink	82006
Duolink PLA probe anti-Rabbit MINUS	Olink	82005
Duolink Ligation solution	Olink	82009
Duolink Amplification Red solution	Olink	82018
T4 DNA ligase	Thermo Scientific	EL0016
Phi29 DNA polymerase	Thermo Scientific	4002
Recombinant Human PDGF-BB	Peprotech	100-14B
**Plates and flasks**		
175 cm2 CellBIND™ Surface Cell Culture Flasks	Corning Life Sciences	3292
Nunc MaxiSorp plates	Thermo Fisher Scientifc	44-2404-21
Black, non-binding surface, flat bottom 96-well plates	Corning Life Sciences	3993
Tissue culture Dish 100mm	Sarstedt	83.3902
Nunc™ Lab-Tek™ II CC2™ Chamber Slide System	Sigma	S6815
MRC 2 Well Crystallization Plate in UVXPO	Hampton Research	HR3-106
**Software**		
MaxQuant	2.0.1.0	https://www.maxquant.org/ https://doi.org/10.1038/nbt.1511
Perseus	2.0.3.0	https://www.maxquant.org/perseus/ https://doi.org/10.1038/nmeth.3901
Cellprofiler	3.0.0	https://cellprofiler.org/previous-releases
Leica Application Suit X software		Leica

Cytoscape	v.3.9.1	https://cytoscape.org/download.html
GraphPad Prism	v9.3.1	https://www.graphpad.com/scientific-software/prism/
Pymol	v2.3.5	https://pymol.org/2/
**Instruments and other**		
iD5 plate reader	Molecular Devices	
iD5 plate reader	Molecular Devices	
Concentrator Plus	Eppendorf	
Illumina MiSeq v3	Illumina	MS-102-3001
Syringe filter, Filtropur S	Sarstedt	83.1826.001
Easy-nLC 1000	Thermo Scientific	
Zen 2 (Blue edition)	Zeiss	
Acclaim PepMap 100 pre-column	Thermo Scientific	164535
PepMap RSLC C18 analytical column	Thermo Scientific	164534
Q Exactive Plus	Thermo Scientific	
TROPHOS Plate RUNNER HD®	Dioscure, Marseille, France	
Plate washer	Molecular devices	
Odyssey CLx Imaging system	LI-COR	9140
MALDI TOF/MS Ultraflex III	Bruker	
Diamond Light Source		Didcot, UK
Zeiss Imager Z2	Zeiss	

References

1. Markova, B., Herrlich, P., Ronnstrand, L. & Bohmer, F.D. Identification of protein tyrosine phosphatases associating with the PDGF receptor. *Biochemistry* **42**, 2691-9 (2003).
2. Tenev, T. et al. Perinuclear localization of the protein-tyrosine phosphatase SHP-1 and inhibition of epidermal growth factor-stimulated STAT1/3 activation in A431 cells. *Eur J Cell Biol* **79**, 261-71 (2000).
3. Asghar, N. et al. The role of the poly(A) tract in the replication and virulence of tick-borne encephalitis virus. *Sci Rep* **6**, 39265 (2016).
4. Islam, M.K. et al. Anti-Rift Valley fever virus activity in vitro, pre-clinical pharmacokinetics and oral bioavailability of benzavir-2, a broad-acting antiviral compound. *Sci Rep* **8**, 1925 (2018).
5. Niedrig, M. et al. Monoclonal antibodies directed against tick-borne encephalitis virus with neutralizing activity in vivo. *Acta Virol* **38**, 141-9 (1994).
