# Supplementary material for: Large-scale phage-based screening reveals extensive pan-viral mimicry of host short linear motifs
Source: Nat Commun. 2023 Apr 26;14:2409. doi: 10.1038/s41467-023-38015-5 (PMC10132805; doi:10.1038/s41467-023-38015-5)
Supplement: Supplementary file 2 — Description of Additional Supplementary Files [file 41467_2023_38015_MOESM2_ESM.pdf]

Title: Supplementary data 1.
Description: RiboVD library design and sequenced coverage.

Title: Supplementary data 2.
Description: Table of expression constructs and information on proteins screened.

Title: Supplementary data 3.
Description: RiboVD selection results. Information is provided on bait protein used, confidence of interaction, peptide found, annotation on host protein, viral species and viral family.

Title: Supplementary data 4.
Description: RiboVD motif benchmarking set.

Title: Supplementary data 5.
Description: RiboVD protein-protein interaction benchmarking set.

Title: Supplementary data 6.
Description: Co-occurrence of viral SLiMs targeting human bait-bait pairs.

Title: Supplementary data 7.
Description: GO term enrichment analysis of expanded host-virus network.

Title: Supplementary data 8.
Description: Overview of peptides used for affinity measurements together with resulting affinity data.

Title: Supplementary data 9.
Description: Details of constructs used for cell based experiments.

Title: Supplementary data 10.
Description: AP-MS data.
