# Supplementary material for: Large-scale phage-based screening reveals extensive pan-viral mimicry of host short linear motifs
Source: Nat Commun. 2023 Apr 26;14:2409. doi: 10.1038/s41467-023-38015-5 (PMC10132805; doi:10.1038/s41467-023-38015-5)
Supplement: Supplementary file 3 — Reporting Summary [file 41467_2023_38015_MOESM3_ESM.pdf]

nature portfolio

Corresponding author(s): Ylva Ivarsson, Per Jemth, Norman Davey

Last updated by author(s): 10/3/2023

Reporting Summary

Nature Portfolio wishes to improve the reproducibility of the work that we publish. This form provides structure for consistency and transparency in reporting. For further information on Nature Portfolio policies, see our Editorial Policies and the Editorial Policy Checklist.

Statistics

For all statistical analyses, confirm that the following items are present in the figure legend, table legend, main text, or Methods section.

n/a	Confirmed	
☐	☒	The exact sample size (*n*) for each experimental group/condition, given as a discrete number and unit of measurement
☐	☒	A statement on whether measurements were taken from distinct samples or whether the same sample was measured repeatedly
☐	☒	The statistical test(s) used AND whether they are one- or two-sided<br>*Only common tests should be described solely by name; describe more complex techniques in the Methods section.*
☒	☐	A description of all covariates tested
☒	☐	A description of any assumptions or corrections, such as tests of normality and adjustment for multiple comparisons
☐	☒	A full description of the statistical parameters including central tendency (e.g. means) or other basic estimates (e.g. regression coefficient) AND variation (e.g. standard deviation) or associated estimates of uncertainty (e.g. confidence intervals)
☐	☒	For null hypothesis testing, the test statistic (e.g. *F*, *t*, *r*) with confidence intervals, effect sizes, degrees of freedom and *P* value noted<br>*Give P values as exact values whenever suitable.*
☒	☐	For Bayesian analysis, information on the choice of priors and Markov chain Monte Carlo settings
☒	☐	For hierarchical and complex designs, identification of the appropriate level for tests and full reporting of outcomes
☒	☐	Estimates of effect sizes (e.g. Cohen's *d*, Pearson's *r*), indicating how they were calculated

Our web collection on statistics for biologists contains articles on many of the points above.

Software and code

Policy information about availability of computer code

Data collection	NGS sequencing data collection was performed using Illumina MySeq v3.<br>For PLA and cell surface fluorescence experiments: Software for image acquisition was Zen Blue 2 (Zeiss).<br>FP data was recorded using iD5 plate reader (Molecular Devices).<br>MS data was recorded using a MALDI TOF/MS Ultraflex III.<br>x-ray data was recorded using the Diamond Light Source.
Data analysis	NGS primary data processing was done as described before (Benz et al., 2022) with custom python scripts (available at: https://bitbucket.org/daveylab/phage_display_pipeline/).<br>For PLA and cell surface fluorescence experiments: CellProfiler v. 3.0.0 pipeline is described in the methods section.<br>For AP-MS: MaxQuant (2.0.1.0) and Perseus (2.0.3.0)<br>For image analysis: Leica Application Suit X software<br>For affinity measurement data analysis: GraphPad Prism (v9.3.1)<br>For structural analysis and visualization: Pymol (v2.3.5)<br>Bioinformatic analysis: python > 3.8.8, networkx 2.5, numpy 1.22.3, scipy 1.6.3, sklearn 0.24,<br>Matplotlib 3.4.2, seaborn 0.11.1, Pandas 1.2.4, Nimfa 1.4, igraph<br>0.9.10, cytoscape 3.9, PepTools (as in Benz et al., 2022; avilable at: http://slim.icr.ac.uk/tools/peptools/)

For manuscripts utilizing custom algorithms or software that are central to the research but not yet described in published literature, software must be made available to editors and reviewers. We strongly encourage code deposition in a community repository (e.g. GitHub). See the Nature Portfolio guidelines for submitting code & software for further information.

Data

Policy information about availability of data

All manuscripts must include a data availability statement. This statement should provide the following information, where applicable:

- Accession codes, unique identifiers, or web links for publicly available datasets
- A description of any restrictions on data availability
- For clinical datasets or third party data, please ensure that the statement adheres to our policy

The mass spectrometry proteomics data have been deposited to the ProteomeXchange Consortium via the PRIDE (Perez-Riverol et al., (2022)) partner repository with the dataset identifier PXD033874. The crystal structures have been deposited in PDB and are available with the PDBis 7BN1, 7BN2 and 7BN3. The interaction data has been deposited to IntAct with the identifier IM-29580. Relevant code has been made available (https://zenodo.org/deposit/6583610).

Human research participants

Policy information about studies involving human research participants and Sex and Gender in Research.

Reporting on sex and gender	Not relevant
Population characteristics	Not relevant
Recruitment	Not relevant
Ethics oversight	Not relevant

Note that full information on the approval of the study protocol must also be provided in the manuscript.

Field-specific reporting

Please select the one below that is the best fit for your research. If you are not sure, read the appropriate sections before making your selection.

☒ Life sciences ☐ Behavioural & social sciences ☐ Ecological, evolutionary & environmental sciences

For a reference copy of the document with all sections, see nature.com/documents/nr-reporting-summary-flat.pdf

Life sciences study design

All studies must disclose on these points even when the disclosure is negative.

Sample size	No sample size calculation was performed using statistical methods. Sample sizes were chosen based on similar studies performed previously.<br>For PLA and cell surface fluorescence experiments: Each experiment was replicated three to six times. For each experiment three images per condition were acquired, resulting in at least nine images being analyzed for each condition and experiment. Data was analyzed on a per-cell basis. The number of cells needed for analysis was not predetermined, and varies between experiment as it depended on the number cells in frame in the microscopy images. Sample size in number of cells analyzed are provided in the figure legends.
Data exclusions	Phage display: The analysis of the phage display data was focused on medium/high confidence peptides, as defined using previously established quality metrics (see Benz et al., 2022). Low confidence peptides were excluded from the analysis.<br>For PLA and cell surface fluorescence experiments: No data was excluded from the analysis.<br>For affinity measurements: No data was excluded as long as the peptides used passed the quality control (that is, being free from fluorescent contaminants).<br>Other than that, no data was excluded as long as the positive/negative controls were working.
Replication	Our experimental results were reliably reproduced through repeated experiments. The number of independent replicates and data points are indicated in the figure legends and the method section.<br>Phage display selections were performed in triplicates (at least).<br>For PLA and cell surface fluorescence experiments: Each experiment was replicated at least three times.
Randomization	We did not randomize the experiments. Randomization was judged not possible/necessary for the experiments included in this work.<br>For phage display selections: protein baits were randomly distributed in 96 well plates.<br>For PLA and cell surface fluorescence experiments: For each experiment, images were acquired from three random regions of the well.
Blinding	Blinding was generally not applied/judged not necessary.<br>Phage selections; All samples and negative controls were treated equally during the selections.<br>For PLA and cell surface fluorescence experiments: Blinding was not applicable to the study.

Reporting for specific materials, systems and methods

We require information from authors about some types of materials, experimental systems and methods used in many studies. Here, indicate whether each material, system or method listed is relevant to your study. If you are not sure if a list item applies to your research, read the appropriate section before selecting a response.

Materials & experimental systems

n/a	Involved in the study
☐	☒ Antibodies
☐	☒ Eukaryotic cell lines
☒	☐ Palaeontology and archaeology
☒	☐ Animals and other organisms
☒	☐ Clinical data
☒	☐ Dual use research of concern

Methods

n/a	Involved in the study
☒	☐ ChIP-seq
☒	☐ Flow cytometry
☒	☐ MRI-based neuroimaging

Antibodies

Antibodies used

Goat-anti-FLAGtag, abcam,ab1257; Mouse-anti-FLAG M2, Sigma Aldrich, F1804; mouse-anti-clathrin, abcam, ab2731; Goat-anti-GST, Cytiva, 274577012; rabbit-anti-PDGFR, Cell Signaling Technology, #3169; mouse-anti-PDGFRβ-pY751, Cell Signaling Technology, #3166; Donkey anti-Goat IgG (H+L) Highly Cross-Adsorbed Secondary Antibody, Alexa Fluor Plus 647, Invitrogen, A32849; Donkey anti-Rabbit IgG (H+L) Highly Cross-Adsorbed Secondary Antibody, Alexa Fluor Plus 555, Invitrogen, A32794; goat-anti PDGFRβ, RnD Systems, AF385; rabbit-anti-FLAGtag, Cell Signaling Technology, #14793S; rabbit anti-SARS-CoV-2 Nucleocapsid, Sino Biological Inc, 40143-R001; mouse anti-Flavivirus Group Antigen Antibody, clone D1-4G2-4-15, ATCC, HB-112; mouse anti-YFV E, ATCC, CRL 1689; mouse anti dsRNA J2, Scicons, 10010500; rabbit anti-VSV-G, Sigma, V4888-200UG; donkey anti-Rabbit IgG (H+L) Highly Cross-Adsorbed Secondary Antibody, Alexa Fluor 555, Invitrogen, A31572; donkey anti-Mouse IgG (H+L) Highly Cross-Adsorbed Secondary Antibody, Alexa Fluor 555, Invitrogen, A31570; Goat anti-Rabbit IgG (H+L) Secondary Antibody, HRP, Invitrogen, 31460; Goat anti-Mouse IgG (H+L) Secondary Antibody, HRP, Invitrogen, 31430; IRDye® 680RD Donkey anti-Goat IgG secondary Antibody, LI-COR, 925-68074; IRDye® 800CW Goat anti-Mouse IgG Secondary Antibody, LI-COR, 925-32210; mouse anti-TBEV E 19/1786, PMID: 7817895

Validation

All antibodies have been validated by the manufacturers, in other publications or in house using established protocols.

For western blots of pulldowns: Mouse-anti-FLAG M2 and Goat-anti-GSTantibodies were directly purchased from manufacturers and were validated by the manufacturers.

For PLA and cell surface fluorescence experiments: All primary antibodies were validated in house with Western blotting and immunofluorescence. All primary antibodies have also been validated with Western blotting by the respective manufacturers. Goat-anti-FLAGtag (ab1257, abcam) was additionally indicated for use in ELISA, immunocytochemistry and immunohistochemistry by the manufacturer. Mouse-anti-clathrin (ab2731, abcam) was additionally validated with immunohistochemistry by the manufacturer. Rabbit-anti-PDGFRβ (#3169, Cell Signaling Technology) was additionally validated with immunoprecipitation and immunohistochemistry by the manufacturer. Mouse-anti-PDGFRβ-pY751 (#3166, Cell Signaling Technology) was additionally validated with immunoprecipitation by the manufacturer. Goat-anti-PDGFRβ (AF385, RnD Systems) was additionally validated with flow cytometry and immunohistochemistry by the manufacturer. Rabbit-anti-FLAG (#14793S, Cell Signaling Technology) was additionally validated with immunoprecipitation, immunohistochemistry, immunofluorescence, flow cytometry and ChIP by the manufacturer.

For viral assayss:SARS-CoV-2 (2019-nCoV) Nucleocapsid Antibody, Rabbit Mab, validated by company and have been used in many other publiations. Mouse Anti-Flavivirus Envelope Protein Antibody (4G2) validated by company and used in many other publications. Mouse anti-YFV E CRL1689 validated by company and used in many other publications. Mouse anti double-stranded RNA (J2). The J2 anti-dsRNA IgG2a monoclonal antibody has become the gold standard in dsRNA detection. Validated by company and used in many other publications. Anti-VSV-G antibody produced in rabbit. Validated by company.

Eukaryotic cell lines

Policy information about cell lines and Sex and Gender in Research

Cell line source(s)

HEK293: Sigma (85120602)
Lenti-X 293T: Takara Bio (Z2180N (632180)

VeroB4: Kind gift of Gerhard Dobler Bundeswehr Institute of Microbiology, Munich, Germany.
VeroE6: Kind gift of Mattias Forsell Umeå University

For PLA and cell surface fluorescence experiments: HEK293 was a kind gift from Prof. Aristidis Moustakas, Uppsala University, Sweden. HEK293-PDGFRβ-HA was a kind gift from Prof. Frank-D. Böhmer, Friedrich Schiller University, Jena, Germany (PMID: 12614164, PMID: 10826494).

Authentication	Cell lines were authenticated by the manufacturers. None of the cell lines were in-house authenticated.
Mycoplasma contamination	Lenti-X 293T cell lines has been tested and found to be free from Mycoplasma by the manufacturer.<br>For PLA and cell surface fluorescence experiments: All cell lines were tested negative for mycoplasma.<br>For viral assays: All cell lines were tested negative for mycoplasma.
Commonly misidentified lines<br>(See ICLAC register)	No commonly misidentified lines according to the ICLAC register were used.
